# Correction: Genomic basis for drought resistance in European beech forests threatened by climate change

**DOI:** 10.7554/eLife.102872

**Published:** 2024-09-06

**Authors:** Markus Pfenninger, Friederike Reuss, Angelika KIebler, Philipp Schönnenbeck, Cosima Caliendo, Susanne Gerber, Berardino Cocchiararo, Sabrina Reuter, Nico Blüthgen, Karsten Mody, Bagdevi Mishra, Miklós Bálint, Marco Thines, Barbara Feldmeyer

**Keywords:** Other

Pfenninger M, Reuss F, Kiebler A, Schönnenbeck P, Caliendo C, Gerber S, Cocchiararo B, Reuter S, Blüthgen N, Mody K, Mishra B, Bálint M, Thines M, Feldmeyer B. 2021. Genomic basis for drought resistance in European beech forests threatened by climate change. *eLife* **10**:e65532. doi: 10.7554/eLife.65532.

Published 16 June 2021

A concern was raised by a reader regarding the soundness of the presented Linear Discriminant Analysis (LDA) for predicting drought-resistant phenotypes from genotypes at candidate loci. Specifically, it was criticised that we performed an LDA without an independent test set and presented the model fit as a result. We acknowledge that this likely lead to model overfitting, given the large number of degrees of freedom. During the initial review process, we followed the original reviewer’s comments and acknowledged the potential overfitting of our method in both our response and the final paper version. Additionally, we included an analysis using a Machine Learning algorithm for feature selection, specifically the non-parametric entropy-based Scalable Probabilistic Analysis (eSPA*), which employs a cross-validation approach. This involved 100 independent runs with 75% of the samples used for training and 25% for the test-set. In response to the justified critique of the LDA approach, we have removed it from the corrected version. Instead, we refer to the more robust eSPA* analysis in the results and interpretation. Please note that we have used the latest version of the eSPA* approach, which was not available at the time of the initial publication.

Abstract

Corrected text:

A non-parametric machine learning approach on 98 validation samples yielded 20 informative loci which allowed an 88% prediction probability of the drought phenotype.

Original text:

A SNP-assay with 70 loci allowed predicting drought phenotype in 98.6% of a validation sample of 92 trees.

Results

Corrected text:

We applied a non-parametric Machine Learning algorithm for simultaneous feature selection and clustering that was especially designed for small sample sizes (Gerber et al., 2020, Horenko, 2020). By selecting the 20 most informative SNPs, the method identified four different clusters. Forty-seven resistant trees were correctly assigned to cluster 1 and 2, while nine susceptible trees were falsely allocated there. Thirty-nine susceptible trees were correctly assigned in clusters 3 and 4, while three resistant trees also fell in these categories. In this way, 88% of the 98 trees could be correctly classified according to their observed phenotype.

Original text:

Linear discriminant analysis (LDA) correctly predicted the observed phenotype from the genotype in 91 of 92 cases (98.9%). Prediction success decreased to 65% when successively removing loci from the analysis (Figure 3—figure supplement 5). Nevertheless, ordering the individuals according to the LDA score of axis 1 revealed no clear genotype pattern that distinguished healthy from damaged trees (Figure 3C). Observed heterozygosity at loci used in the SNP assay of individuals in the upper half of predictive values for a healthy phenotype was not significantly different from heterozygosity of the lower half (Figure 3—figure supplement 6). Ordering the loci according to their squared loadings showed that loci’s contribution to the genomic prediction differed substantially (Figure 5). As expected, the histogram of LDA scores showed two peaks, corresponding to the two phenotypes (Figure 3—figure supplement 7).

To validate the results of the LDA prediction and to circumvent potential overfitting due to the small sample size, we also applied a non-parametric Machine Learning algorithm for feature selection and clustering that was especially designed for small sample sizes (Gerber et al., 2020; Horenko, 2020). The method identified the 20 most-significant SNPs allowing to make an almost 85% correct classification that distinguished healthy from damaged trees (Supplementary file 1G).

Discussion

Corrected text:

We achieved a high level of accuracy using genomic data to statistically predict the drought phenotype from individuals not used to identify drought-associated SNP loci. We used a non-parametric machine learning algorithm that has been shown to produce robust results, especially for small sample sizes (Horenko, 2020). Please note that the method is not trying to causally and quantitatively explain phenotypic differences, but uses statistical associations for prediction. The analysis confirmed that we mainly identified alleles widespread throughout the sampled range and not locally specific.

Original text:

We achieved a high level of accuracy using genomic data to predict the drought phenotype from individuals not used to identify drought-associated SNP loci. However, due to the small sample size, LDA might have resulted in overfitting (Hawkins, 2004). We therefore also used a non-parametric machine learning algorithm that has been shown to produce more robust results, especially for small sample sizes (Horenko, 2020). Both analyses confirmed that we mainly identified alleles widespread throughout the sampled range and not locally specific.

Material and methods

Corrected text:

To predict drought susceptibility from genotype data, we used a non-parametric entropy-based Scalable Probabilistic Analysis framework (eSPA*, Vecchi et al., 2022). This method allows simultaneous solution of feature selection and clustering problems, meaning that does not rely on a particular choice of user-defined parameters and has been shown to produce more robust results than most other machine learning classification algorithms, especially for small sample sizes (Gerber et al., 2020, Horenko, 2020). For the optimization process, eSPA* calculates a probability for each beech to belong to one of k clusters, with each cluster representing a specific combination of genotypes at SNP sites. Each cluster in turn has a specific probability of including either drought-resistant, or drought sensitive beeches. If a beech is assigned to e.g. cluster 1 (pattern 1) based on its specific SNP profile this means that this beech has at least a 79% Likelihood to be drought resistant. All beech trees assigned to a certain cluster are also given a probability indicating how "well" this beech fits to this cluster. This is estimated using a distance function that calculates the goodness of fit to the cluster. We applied a cross-validation approach with 100 independent runs of 75% of the samples as training and 25% of the samples as test-set. By iterating this process over several number of clusters, the optimal solution is found.

Original text:

To predict drought susceptibility from genotype data, we used an LDA on 92 genotypes scored with the Fluidigm assay at 70 loci. Genotypes homozygous for the reference allele were scored as 0, heterozygous as 1 and homozygous alternate alleles as 2. We used the LDA option implemented in PAST v. 4.05. (Hammer et al., 2001).

We also used a non-parametric entropy-based Scalable Probabilistic Analysis framework (eSPA). This method allows simultaneous solution of feature selection and clustering problems, meaning that does not rely on a particular choice of user-defined parameters and has been shown to produce more robust results, especially for small sample sizes (Gerber2020, Horenko2020). Following the suggestion of the user manual, eSPA analysis was run 100 times with independent cross-validations of the Area Under the Curve (AUC) on the validation data.

References:

Inserted Reference:

Vecchi E, Pospíšil L, Albrecht S, O’Kane TJ, Horenko I. 2022. eSPA+: Scalable Entropy-Optimal Machine Learning Classification for Small Data Problems. *Neural Computation* **34**:1220–1255. 10.1162/neco_a_01490, 35344997

Deleted Reference:

Hawkins DM. 2004. The problem of overfitting. *Journal of Chemical Information and Computer Sciences* **44**:1–12. 10.1021/ci0342472, 14741005

The corrected Figure 3 is shown here:

**Figure:**
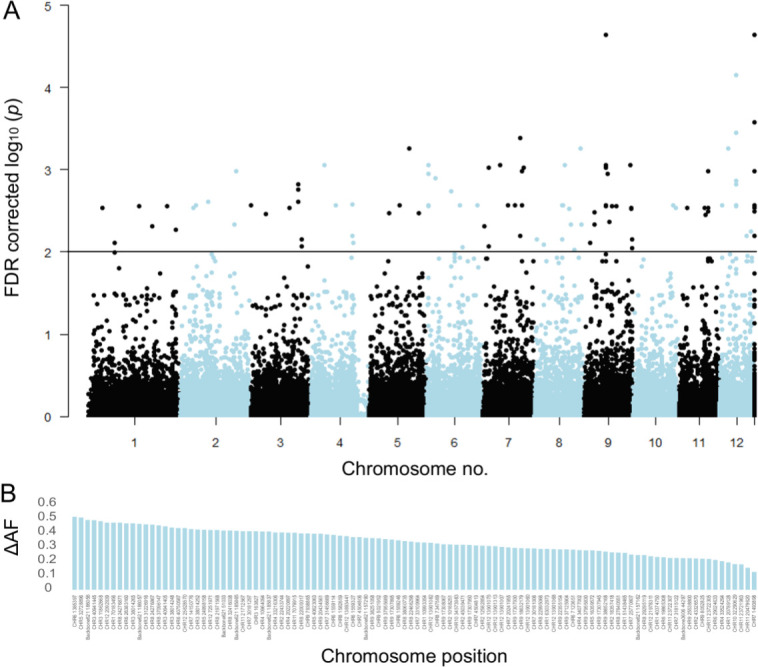


The originally published Figure 3 is shown for reference:

**Figure:**
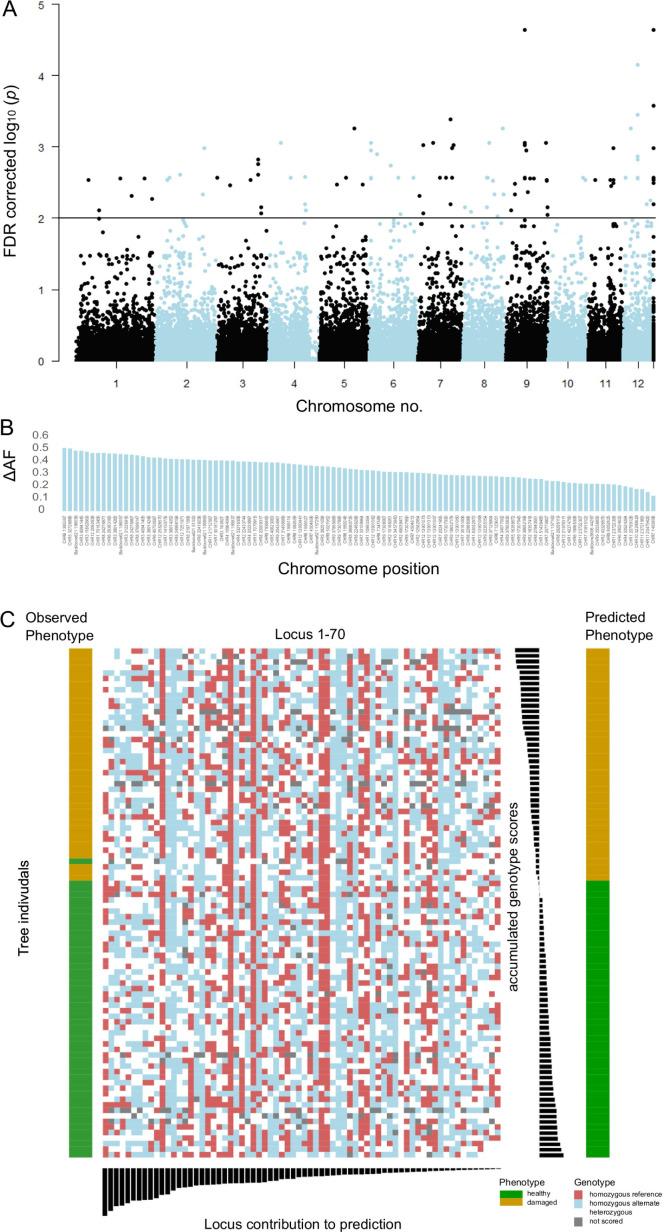


The following figure supplements and figure supplement legends have been removed:

Figure 3—figure supplement 5. Increase of LDA prediction success with the number of loci involved. Loci were added according to their decreasing contribution in the final analysis.

Figure 3—figure supplement 6. Comparison of observed heterozygosity between lower and upper half of predictive values “healthy” in DA. The medians are not significantly different (Mann-Whitney U=293.5, p same median = 0.72).

Figure 3—figure supplement 7. Histogram of LDA results. Individuals, where the predicted phenotype did not match the observed phenotype are shown in grey, individuals with matching observed/predicted phenotype in green (healthy) or in ochre (damaged).

The originally published Figure 3—figure supplement 5 is shown for reference:

**Figure:**
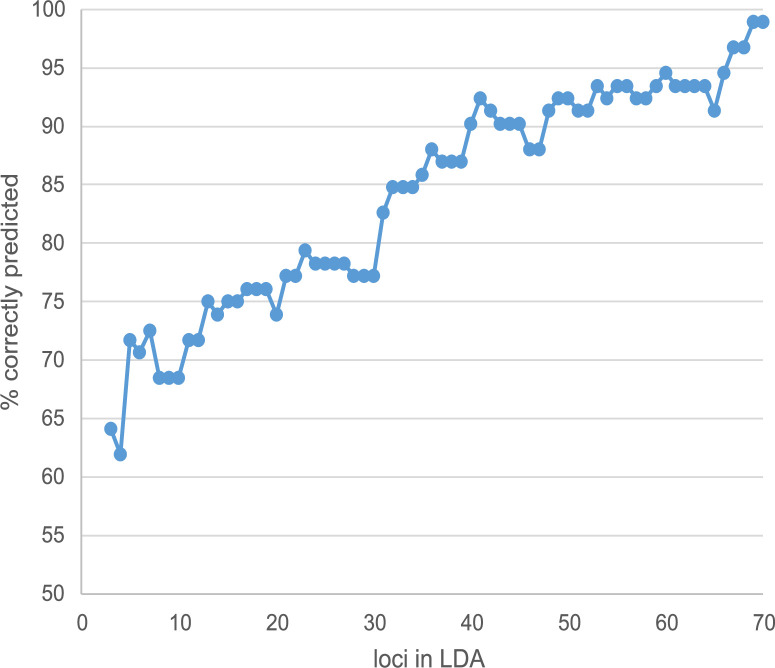


The originally published Figure 3—figure supplement 6 is shown for reference:

**Figure:**
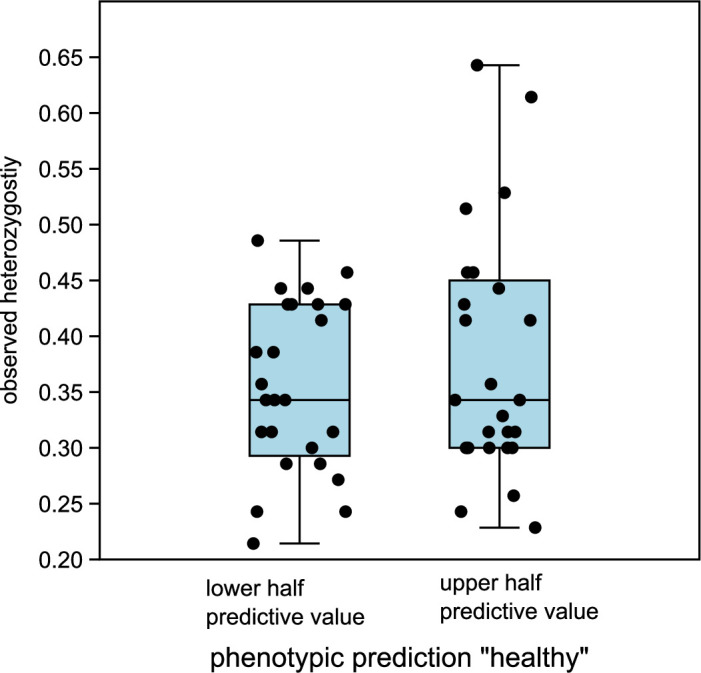


The originally published Figure 3—figure supplement 7 is shown for reference:

**Figure:**
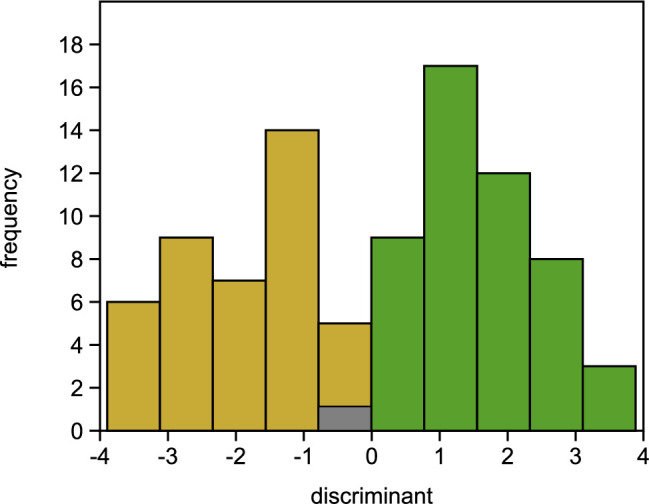


Corrected Data availability:

Sequencing data have been deposited at ENA under project code PRJEB41889. The genome assembly including the annotation is available under the accession PRJNA450822. eSPA* input-file for MatLab, containing both source data and source code is available as Source code 1.

Originally published Data availability statement is shown for reference:

Sequencing data have been deposited at ENA under project code PRJEB41889. The genome assembly including the annotation is available under the accession PRJNA450822.

The following legend has been added for Source code 1:

Source code 1. eSPA* input-file for MatLab.

The article has been corrected accordingly.

