# Supplementary material for: Genomic basis for drought resistance in European beech forests threatened by climate change
Source: eLife. 2021 Jun 16;10:e65532. doi: 10.7554/eLife.65532 (PMC8266386; doi:10.7554/eLife.65532)
Supplement: Supplementary file 1. — (A) Sampled Fagus sylvatica individuals. (B) Results from Mann–Whitney U-test on difference between damaged and healthy trees for various parameters. (C) List of genes with significant single-nucleotide polymorphisms (SNPs). (D) List of genes closest to significant SNPs. (E) List of 20 most informative SNPs as selected by the entropy-based Scalable Probabilistic Analysis (eSPA) allowing for 85% correct classification. (F) Software pipeline and commands used for PoolSeq analysis. (G) Workflow individual reseq GWAS. [file elife-65532-supp1.docx]

## **Supplemental File 1**

**Supplementary file 1A**. Sampled *Fagus sylvatica* individuals. Given are the phenotype, sampling location, sampling date, geographical coordinates in decimal degrees, the pool to which the individual contributed to (h=healthy, d=damaged), whether it was individually re-sequenced (x) and whether it was used in the SNP assay to validate the results (x).

| TreeID | Phenotype | Location | Sampling date | Latitude | Longitude | Pool  (set 1) | individually re-sequenced | Validation  (set 2) |
| --- | --- | --- | --- | --- | --- | --- | --- | --- |
| PF_001 | damaged | Königstein | 29.08.2019 | 50.1957342 | 8.4648591 | dNorth |  |  |
| PF_002 | healthy | Königstein | 29.08.2019 | 50.1957187 | 8.4648341 | hNorth | x |  |
| PF_003 | damaged | Königstein | 29.08.2019 | 50.1964726 | 8.4664262 | dNorth | x |  |
| PF_004 | healthy | Königstein | 29.08.2019 | 50.1964232 | 8.4664275 | hNorth | x |  |
| PF_005 | damaged | Königstein | 29.08.2019 | 50.1929421 | 8.456941 | dNorth | x |  |
| PF_006 | healthy | Königstein | 29.08.2019 | 50.1928331 | 8.4568086 | hNorth | x |  |
| PF_007 | damaged | Königstein | 29.08.2019 | 50.1925453 | 8.457816 | dNorth | x |  |
| PF_008 | healthy | Königstein | 29.08.2019 | 50.1925741 | 8.4578839 | hNorth | x |  |
| PF_009 | damaged | Königstein | 29.08.2019 | 50.1926029 | 8.4579518 | dNorth | x |  |
| PF_010 | healthy | Königstein | 29.08.2019 | 50.1926317 | 8.4580197 | hNorth | x |  |
| PF_011 | damaged | Obernhain | 03.09.2019 | 50.2825898 | 8.5712681 | dNorth |  |  |
| PF_012 | healthy | Obernhain | 03.09.2019 | 50.2827911 | 8.5711075 | hNorth |  |  |
| PF_013 | damaged | Obernhain | 03.09.2019 | 50.2820637 | 8.5734056 | dNorth |  |  |
| PF_014 | healthy | Obernhain | 03.09.2019 | 50.282214 | 8.5734601 | hNorth |  |  |
| PF_015 | damaged | Obernhain | 03.09.2019 | 50.2817116 | 8.5761923 | dNorth |  |  |
| PF_016 | healthy | Obernhain | 03.09.2019 | 50.2816381 | 8.5761487 | hNorth | x |  |
| PF_017 | damaged | Obernhain | 03.09.2019 | 50.2821782 | 8.5704083 | dNorth | x |  |
| PF_018 | healthy | Obernhain | 03.09.2019 | 50.2821327 | 8.5704883 | hNorth | x |  |
| PF_019 | damaged | Obernhain | 03.09.2019 | 50.2816941 | 8.5700475 | dNorth | x |  |
| PF_020 | healthy | Obernhain | 03.09.2019 | 50.2817058 | 8.570133 | hNorth | x |  |
| PF_021 | damaged | Usingen | 04.09.2019 | 50.3392732 | 8.4913495 | dNorth | x |  |
| PF_022 | healthy | Usingen | 04.09.2019 | 50.3391762 | 8.491312 | hNorth | x |  |
| PF_023 | damaged | Usingen | 04.09.2019 | 50.3397337 | 8.4924001 | dNorth | x |  |
| PF_024 | healthy | Usingen | 04.09.2019 | 50.3396942 | 8.4924593 | hNorth | x |  |
| PF_025 | damaged | Usingen | 04.09.2019 | 50.344079 | 8.4932305 | dNorth | x |  |
| PF_026 | healthy | Usingen | 04.09.2019 | 50.3440912 | 8.4933098 | hNorth | x |  |
| PF_027 | damaged | Usingen | 04.09.2019 | 50.3452681 | 8.4959808 | dNorth | x |  |
| PF_028 | healthy | Usingen | 04.09.2019 | 50.3451246 | 8.4962431 | hNorth | x |  |
| PF_029 | damaged | Usingen | 04.09.2019 | 50.3416761 | 8.4918323 | dNorth | x |  |
| PF_030 | healthy | Usingen | 04.09.2019 | 50.3417203 | 8.4917173 | hNorth | x |  |
| PF_031 | damaged | Eppenhain | 05.09.2019 | 50.1736813 | 8.3908577 | dNorth | x |  |
| PF_032 | healthy | Eppenhain | 05.09.2019 | 50.1736578 | 8.39113553 | hNorth | x |  |
| PF_033 | damaged | Eppenhain | 05.09.2019 | 50.1750866 | 8.3891472 | dNorth | x |  |
| PF_034 | healthy | Eppenhain | 05.09.2019 | 50.175069 | 8.3892187 | hNorth | x |  |
| PF_035 | damaged | Eppenhain | 05.09.2019 | 50.1754443 | 8.3876407 | dNorth | x |  |
| PF_036 | healthy | Eppenhain | 05.09.2019 | 50.1754007 | 8.3875849 | hNorth | x |  |
| PF_037 | damaged | Eppenhain | 05.09.2019 | 50.1746449 | 8.3872509 | dNorth | x |  |
| PF_038 | healthy | Eppenhain | 05.09.2019 | 50.1746095 | 8.3874874 | hNorth | x |  |
| PF_039 | damaged | Eppenhain | 05.09.2019 | 50.1734334 | 8.3904983 | dNorth | x |  |
| PF_040 | healthy | Eppenhain | 05.09.2019 | 50.1733173 | 8.390211 | hNorth | x |  |
| PF_041 | damaged | Eppenhain | 05.09.2019 | 50.1745915 | 8.3907433 | dNorth | x |  |
| PF_042 | healthy | Eppenhain | 05.09.2019 | 50.1745128 | 8.3908124 | hNorth | x |  |
| PF_050 | damaged | Bad Camberg | 06.09.2019 | 50.2976336 | 8.2980473 | dNorth |  |  |
| PF_051 | healthy | Bad Camberg | 06.09.2019 | 50.2975811 | 8.298169 | hNorth |  |  |
| PF_052 | damaged | Daubringen | 07.09.2019 | 50.6483759 | 8.7419719 | dNorth | x |  |
| PF_053 | healthy | Daubringen | 07.09.2019 | 50.6482664 | 8.7421457 | hNorth | x |  |
| PF_054 | damaged | Daubringen | 07.09.2019 | 50.6480228 | 8.7428739 | dNorth | x |  |
| PF_055 | healthy | Daubringen | 07.09.2019 | 50.6478157 | 8.742954 | hNorth | x |  |
| PF_056 | damaged | Daubringen | 07.09.2019 | 50.648846 | 8.7426283 | dNorth | x |  |
| PF_057 | healthy | Daubringen | 07.09.2019 | 50.6489798 | 8.7423211 | hNorth | x |  |
| PF_058 | damaged | Schwabendorf | 07.09.2019 | 50.8927904 | 8.8879419 | dNorth | x |  |
| PF_059 | healthy | Schwabendorf | 07.09.2019 | 50.8929008 | 8.8881284 | hNorth | x |  |
| PF_060 | damaged | Schwabendorf | 07.09.2019 | 50.8951542 | 8.8822243 | dNorth | x |  |
| PF_061 | healthy | Schwabendorf | 07.09.2019 | 50.8952005 | 8.882144 | hNorth | x |  |
| PF_062 | damaged | Oberurff | 07.09.2019 | 51.0458016 | 9.1505195 | dNorth | x |  |
| PF_063 | healthy | Oberurff | 07.09.2019 | 51.0457499 | 9.1505234 | hNorth | x |  |
| PF_064 | damaged | Oberurff | 07.09.2019 | 51.0461483 | 9.1478112 | dNorth | x |  |
| PF_065 | healthy | Oberurff | 07.09.2019 | 51.0461706 | 9.1474544 | hNorth | x |  |
| PF_066 | damaged | Oberurff | 07.09.2019 | 51.0453396 | 9.1466057 | dNorth | x |  |
| PF_067 | healthy | Oberurff | 07.09.2019 | 51.0453396 | 9.146057 | hNorth | x |  |
| PF_068 | damaged | Oberurff | 07.09.2019 | 51.0463978 | 9.1432389 | dNorth | x |  |
| PF_069 | healthy | Oberurff | 07.09.2019 | 51.0464672 | 9.1435546 | hNorth | x |  |
| PF_070 | damaged | Neukirchen | 08.09.2019 | 50.8848091 | 9.3557289 | dNorth | x |  |
| PF_071 | healthy | Neukirchen | 08.09.2019 | 50.88496 | 9.3560291 | hNorth | x |  |
| PF_072 | damaged | Langenhain | 09.09.2019 | 50.0955818 | 8.4101217 | dNorth | x |  |
| PF_073 | healthy | Langenhain | 09.09.2019 | 50.0956666 | 8.4105235 | hNorth | x |  |
| PF_074 | damaged | Langenhain | 09.09.2019 | 50.0959896 | 8.4104922 | dNorth | x |  |
| PF_075 | healthy | Langenhain | 09.09.2019 | 50.0960509 | 8.4105981 | hNorth | x |  |
| PF_076 | damaged | Langenhain | 09.09.2019 | 50.0947953 | 8.4151317 | dNorth | x |  |
| PF_077 | healthy | Langenhain | 09.09.2019 | 50.0947434 | 8.4150994 | hNorth | x |  |
| PF_078 | damaged | Langenhain | 09.09.2019 | 50.0911467 | 8.41578726 | dNorth | x |  |
| PF_079 | healthy | Langenhain | 09.09.2019 | 50.0912627 | 8.415817 | hNorth |  |  |
| PF_080 | damaged | Langenhain | 09.09.2019 | 50.0901299 | 8.4166724 | dNorth | x |  |
| PF_081 | healthy | Langenhain | 09.09.2019 | 50.090096 | 8.4166748 | hNorth | x |  |
| PF_082 | damaged | Langenhain | 09.09.2019 | 50.0940815 | 8.4100466 | dNorth | x |  |
| PF_083 | healthy | Langenhain | 09.09.2019 | 50.0939183 | 8.4099548 | hNorth | x |  |
| PF_085 | damaged | Theistal | 11.09.2019 | 50.1480865 | 8.2445088 | dNorth | x |  |
| PF_086 | healthy | Theistal | 11.09.2019 | 50.1480164 | 8.2447881 | hNorth | x |  |
| PF_087 | damaged | Theistal | 11.09.2019 | 50.1517267 | 8.2464193 | dNorth | x |  |
| PF_088 | healthy | Theistal | 11.09.2019 | 50.151875 | 8.2461256 | hNorth | x |  |
| PF_089 | damaged | Theistal | 11.09.2019 | 50.1532534 | 8.2456221 | dNorth | x |  |
| PF_090 | healthy | Theistal | 11.09.2019 | 50.1528409 | 8.2455616 | hNorth | x |  |
| PF_091 | damaged | Theistal | 11.09.2019 | 50.1539513 | 8.2466804 | dNorth | x |  |
| PF_092 | healthy | Theistal | 11.09.2019 | 50.1540134 | 8.2462949 | hNorth | x |  |
| PF_093 | damaged | Schlangenbad | 11.09.2019 | 50.1027276 | 8.1136673 | dNorth | x |  |
| PF_094 | healthy | Schlangenbad | 11.09.2019 | 50.1025753 | 8.1136931 | hNorth | x |  |
| PF_095 | damaged | Schlangenbad | 11.09.2019 | 50.0997272 | 8.1101568 | dNorth | x |  |
| PF_096 | healthy | Schlangenbad | 11.09.2019 | 50.099823 | 8.1103387 | hNorth | x |  |
| PF_100 | damaged | Wehrheim | 12.09.2019 | 50.3153263 | 8.5819508 | dNorth | x |  |
| PF_101 | healthy | Wehrheim | 12.09.2019 | 50.315372 | 8.5820711 | hNorth | x |  |
| PF_102 | damaged | Wehrheim | 12.09.2019 | 50.3145513 | 8.5854558 | dNorth | x |  |
| PF_103 | healthy | Wehrheim | 12.09.2019 | 50.3145748 | 8.585572 | hNorth | x |  |
| PF_104 | damaged | Wehrheim | 12.09.2019 | 50.3147404 | 8.585804 | dNorth | x |  |
| PF_105 | healthy | Wehrheim | 12.09.2019 | 50.3146745 | 8.5858773 | hNorth | x |  |
| PF_106 | damaged | Wehrheim | 12.09.2019 | 50.3149149 | 8.5859893 | dNorth | x |  |
| PF_107 | healthy | Wehrheim | 12.09.2019 | 50.31472242 | 8.5861579 | hNorth | x |  |
| PF_108 | damaged | Wehrheim | 12.09.2019 | 50.3149559 | 8.5866045 | dNorth | x |  |
| PF_109 | healthy | Wehrheim | 12.09.2019 | 50.3150733 | 8.5866527 | hNorth | x |  |
| PF_110 | damaged | Wehrheim | 12.09.2019 | 50.3155392 | 8.587186 | dNorth | x |  |
| PF_111 | healthy | Wehrheim | 12.09.2019 | 50.3154613 | 8.5872255 | hNorth | x |  |
| PF_112 | damaged | Wehrheim | 12.09.2019 | 50.3130127 | 8.580265 | dNorth | x |  |
| PF_113 | healthy | Wehrheim | 12.09.2019 | 50.3130103 | 8.5800785 | hNorth | x |  |
| PF_114 | damaged | Wehrheim | 12.09.2019 | 50.3141899 | 8.581059 | dNorth | x |  |
| PF_115 | healthy | Wehrheim | 12.09.2019 | 50.314164 | 8.5809636 | hNorth | x |  |
| S_001 | damaged | Bot. Garten | 26.08.2019 | 49.79971 | 8.95682 | dSouth |  |  |
| S_002 | healthy | Bot. Garten | 26.08.2019 | 49.86852 | 8.67999 | hSouth |  |  |
| S_003 | damaged | Bot. Garten | 26.08.2019 | 49.86856 | 8.68018 | dSouth |  |  |
| S_004 | healthy | Bot. Garten | 26.08.2019 | 49.86856 | 8.68018 | hSouth |  |  |
| S_005 | damaged | Vivarium | 26.08.2019 | 49.86855 | 8.68017 | dSouth |  |  |
| S_006 | healthy | Vivarium | 26.08.2019 | 49.86436 | 8.6842 | hSouth |  |  |
| S_007 | damaged | Vivarium | 26.08.2019 | 49.8644 | 8.68418 | dSouth |  |  |
| S_008 | healthy | Vivarium | 26.08.2019 | 49.8644 | 8.68418 | hSouth |  |  |
| S_009 | damaged | Vivarium | 26.08.2019 | 49.8644 | 8.68418 | dSouth |  |  |
| S_010 | healthy | Vivarium | 26.08.2019 | 49.8644 | 8.68418 | hSouth |  |  |
| S_011 | damaged | Vivarium | 26.08.2019 | 49.8644 | 8.68418 | dSouth |  |  |
| S_012 | healthy | Vivarium | 26.08.2019 | 49.8644 | 8.68418 | hSouth |  |  |
| S_013 | damaged | VivariumII | 26.08.2019 | 49.8644 | 8.68418 | dSouth |  |  |
| S_014 | healthy | VivariumII | 26.08.2019 | 49.8644 | 8.68418 | hSouth |  |  |
| S_015 | damaged | VivariumII | 26.08.2019 | 49.86227 | 8.68876 | dSouth |  |  |
| S_016 | healthy | VivariumII | 26.08.2019 | 49.86241 | 8.68874 | hSouth |  |  |
| S_017 | damaged | VivariumII | 26.08.2019 | 49.86199 | 8.68894 | dSouth |  |  |
| S_018 | healthy | VivariumII | 26.08.2019 | 49.86196 | 8.68885 | hSouth |  |  |
| S_019 | damaged | VivariumII | 26.08.2019 | 49.86092 | 8.69278 | dSouth |  |  |
| S_020 | healthy | VivariumII | 26.08.2019 | 49.86031 | 8.69225 | hSouth |  |  |
| S_021 | damaged | VivariumIII | 27.08.2019 | 49.85883 | 8.69445 | dSouth |  |  |
| S_022 | healthy | VivariumIII | 27.08.2019 | 49.85883 | 8.69445 | hSouth |  |  |
| S_023 | damaged | VivariumIII | 27.08.2019 | 49.85882 | 8.69446 | dSouth |  |  |
| S_024 | healthy | VivariumIII | 27.08.2019 | 49.85882 | 8.69446 | hSouth |  |  |
| S_025 | damaged | VivariumIII | 27.08.2019 | 49.85862 | 8.6944 | dSouth |  |  |
| S_026 | healthy | VivariumIII | 27.08.2019 | 49.85862 | 8.6944 | hSouth |  |  |
| S_027 | damaged | VivariumIII | 27.08.2019 | 49.85637 | 8.69584 | dSouth |  |  |
| S_028 | healthy | VivariumIII | 27.08.2019 | 49.85637 | 8.69584 | hSouth |  |  |
| S_029 | damaged | VivariumIII | 27.08.2019 | 49.8565 | 8.69616 | dSouth |  |  |
| S_030 | healthy | VivariumIII | 27.08.2019 | 49.8565 | 8.69616 | hSouth |  |  |
| S_031 | damaged | VivariumIII | 27.08.2019 | 49.8565 | 8.69616 | dSouth |  |  |
| S_032 | healthy | VivariumIII | 27.08.2019 | 49.8565 | 8.69616 | hSouth |  |  |
| S_033 | damaged | VivariumIII | 27.08.2019 | 49.85576 | 8.69886 | dSouth |  |  |
| S_034 | healthy | VivariumIII | 27.08.2019 | 49.85576 | 8.69886 | hSouth |  |  |
| S_035 | damaged | VivariumIII | 27.08.2019 | 49.85576 | 8.69886 | dSouth |  |  |
| S_036 | healthy | VivariumIII | 27.08.2019 | 49.85576 | 8.69886 | hSouth |  |  |
| S_037 | damaged | VivariumIII | 27.08.2019 | 49.85329 | 8.6938 | dSouth |  |  |
| S_038 | healthy | VivariumIII | 27.08.2019 | 49.85329 | 8.6938 | hSouth |  |  |
| S_039 | damaged | VivariumIII | 27.08.2019 | 49.85329 | 8.6938 | dSouth |  |  |
| S_040 | healthy | VivariumIII | 27.08.2019 | 49.85329 | 8.6938 | hSouth |  |  |
| S_041 | damaged | VivariumIII | 27.08.2019 | 49.85021 | 8.9391 | dSouth |  |  |
| S_042 | healthy | VivariumIII | 27.08.2019 | 49.85021 | 8.9391 | hSouth |  |  |
| S_043 | damaged | Neu-Isenburg | 28.08.2019 | 49.85585 | 8.69554 | dSouth |  |  |
| S_044 | healthy | Neu-Isenburg | 28.08.2019 | 49.85585 | 8.69554 | hSouth |  |  |
| S_045 | damaged | Neu-Isenburg | 28.08.2019 | 49.85585 | 8.69554 | dSouth |  |  |
| S_046 | healthy | Neu-Isenburg | 28.08.2019 | 49.85585 | 8.69554 | hSouth |  |  |
| S_047 | damaged | Neu-Isenburg | 28.08.2019 | 49.85585 | 8.69554 | dSouth |  |  |
| S_048 | healthy | Neu-Isenburg | 28.08.2019 | 49.85585 | 8.69554 | hSouth |  |  |
| S_049 | damaged | Neu-Isenburg | 28.08.2019 | 49.85585 | 8.69554 | dSouth |  |  |
| S_050 | healthy | Neu-Isenburg | 28.08.2019 | 49.85585 | 8.69554 | hSouth |  |  |
| S_051 | damaged | Neu-Isenburg | 28.08.2019 | 49.85585 | 8.69554 | dSouth |  |  |
| S_052 | healthy | Neu-Isenburg | 28.08.2019 | 49.85585 | 8.69554 | hSouth |  |  |
| S_053 | damaged | Neu-Isenburg | 28.08.2019 | 49.85585 | 8.69554 | dSouth |  |  |
| S_054 | healthy | Neu-Isenburg | 28.08.2019 | 49.85585 | 8.69554 | hSouth |  |  |
| S_055 | damaged | Neu-Isenburg | 28.08.2019 | 49.85585 | 8.69554 | dSouth |  |  |
| S_056 | healthy | Neu-Isenburg | 28.08.2019 | 49.85585 | 8.69554 | hSouth |  |  |
| S_057 | damaged | Neu-Isenburg | 28.08.2019 | 49.85585 | 8.69554 | dSouth |  |  |
| S_058 | healthy | Neu-Isenburg | 28.08.2019 | 49.85585 | 8.69554 | hSouth |  |  |
| S_059 | damaged | Neu-Isenburg | 28.08.2019 | 49.85585 | 8.69554 | dSouth |  |  |
| S_060 | healthy | Neu-Isenburg | 28.08.2019 | 49.85585 | 8.69554 | hSouth |  |  |
| S_061 | damaged | Westwald | 29.09.2019 | 49.855069 | 8.617017 | dSouth |  |  |
| S_062 | healthy | Westwald | 29.09.2019 | 49.855069 | 8.617017 | hSouth |  |  |
| S_063 | damaged | Westwald | 29.09.2019 | 49.85495 | 8.61703 |  |  | x |
| S_064 | healthy | Westwald | 29.09.2019 | 49.85495 | 8.61703 |  |  |  |
| S_065 | damaged | Westwald | 29.09.2019 | 49.85495 | 8.61703 |  |  |  |
| S_066 | healthy | Westwald | 29.09.2019 | 49.85495 | 8.61703 |  |  | x |
| S_067 | damaged | Westwald | 29.09.2019 | 49.85494 | 8.61702 | dSouth |  |  |
| S_068 | healthy | Westwald | 29.09.2019 | 49.85494 | 8.61702 | hSouth |  |  |
| S_069 | damaged | Westwald | 29.09.2019 | 49.854909 | 8.618146 | dSouth |  |  |
| S_070 | healthy | Westwald | 29.09.2019 | 49.854909 | 8.618146 | hSouth |  |  |
| S_071 | damaged | Westwald | 29.09.2019 | 49.85494 | 8.61702 |  |  | x |
| S_072 | healthy | Westwald | 29.09.2019 | 49.85494 | 8.61702 |  |  | x |
| S_073 | damaged | Westwald | 29.09.2019 | 49.85494 | 8.61702 | dSouth |  |  |
| S_074 | healthy | Westwald | 29.09.2019 | 49.85494 | 8.61702 | hSouth |  |  |
| S_075 | damaged | Westwald | 29.09.2019 | 49.85494 | 8.61702 | dSouth |  |  |
| S_076 | healthy | Westwald | 29.09.2019 | 49.85494 | 8.61702 | hSouth |  |  |
| S_077 | damaged | Westwald | 29.09.2019 | 49.85494 | 8.61702 |  |  | x |
| S_078 | healthy | Westwald | 29.09.2019 | 49.85494 | 8.61702 |  |  | x |
| S_079 | damaged | Westwald | 29.09.2019 | 49.85494 | 8.61702 |  |  |  |
| S_080 | healthy | Westwald | 29.09.2019 | 49.85494 | 8.61702 |  |  |  |
| S_081 | damaged | Westwald | 29.09.2019 | 49.85494 | 8.61702 |  |  | X |
| S_082 | healthy | Westwald | 29.09.2019 | 49.85494 | 8.61702 |  |  | X |
| S_083 | damaged | Westwald | 29.09.2019 | 49.85494 | 8.61702 |  |  |  |
| S_084 | healthy | Westwald | 29.09.2019 | 49.85494 | 8.61702 |  |  |  |
| S_085 | damaged | Roßdorf | 02.09.2019 | 49.85494 | 8.61702 | dSouth |  |  |
| S_086 | healthy | Roßdorf | 02.09.2019 | 49.85494 | 8.61702 | hSouth |  |  |
| S_087 | damaged | Roßdorf | 02.09.2019 | 49.85943 | 8.70791 |  |  | X |
| S_088 | healthy | Roßdorf | 02.09.2019 | 49.85943 | 8.70791 |  |  | X |
| S_089 | damaged | Roßdorf | 02.09.2019 | 49.85943 | 8.70786 | dSouth |  |  |
| S_090 | healthy | Roßdorf | 02.09.2019 | 49.85943 | 8.70786 | hSouth |  |  |
| S_091 | damaged | Roßdorf | 02.09.2019 | 49.85977 | 8.70795 | dSouth |  |  |
| S_092 | healthy | Roßdorf | 02.09.2019 | 49.85977 | 8.70795 | hSouth |  |  |
| S_093 | damaged | Roßdorf | 02.09.2019 | 49.86221 | 8.718 |  |  | X |
| S_094 | healthy | Roßdorf | 02.09.2019 | 49.86221 | 8.718 |  |  | X |
| S_095 | damaged | Roßdorf | 02.09.2019 | 49.86211 | 8.71801 | dSouth |  |  |
| S_096 | healthy | Roßdorf | 02.09.2019 | 49.86211 | 8.71801 | hSouth |  |  |
| S_097 | damaged | Roßdorf | 02.09.2019 | 49.86211 | 8.71801 | dSouth |  |  |
| S_098 | healthy | Roßdorf | 02.09.2019 | 49.86211 | 8.71801 | hSouth |  |  |
| S_099 | damaged | Roßdorf | 02.09.2019 | 49.86089 | 8.70835 |  |  |  |
| S_100 | healthy | Roßdorf | 02.09.2019 | 49.86089 | 8.70835 |  |  |  |
| S_101 | damaged | Roßdorf | 02.09.2019 | 49.86095 | 8.70771 |  |  |  |
| S_102 | healthy | Roßdorf | 02.09.2019 | 49.86095 | 8.70771 |  |  |  |
| S_103 | damaged | Roßdorf | 02.09.2019 | 49.86103 | 8.70769 |  |  | x |
| S_104 | healthy | Roßdorf | 02.09.2019 | 49.86103 | 8.70769 |  |  | x |
| S_105 | damaged | Burg Breuberg | 03.09.2019 | 49.8259 | 9.0341 | dSouth |  |  |
| S_106 | healthy | Burg Breuberg | 03.09.2019 | 49.8259 | 9.0341 | hSouth |  |  |
| S_107 | damaged | Burg Breuberg | 03.09.2019 | 49.82447 | 9.03196 | dSouth |  |  |
| S_108 | healthy | Burg Breuberg | 03.09.2019 | 49.82447 | 9.03196 | hSouth |  |  |
| S_109 | damaged | Burg Breuberg | 03.09.2019 | 49.82457 | 9.03155 | dSouth |  |  |
| S_110 | healthy | Burg Breuberg | 03.09.2019 | 49.82457 | 9.03155 | hSouth |  |  |
| S_111 | damaged | Burg Breuberg | 03.09.2019 | 49.82736 | 9.0267 | dSouth |  |  |
| S_112 | healthy | Burg Breuberg | 03.09.2019 | 49.82736 | 9.0267 | hSouth |  |  |
| S_113 | damaged | Burg Breuberg | 03.09.2019 | 49.827 | 9.02706 | dSouth |  |  |
| S_114 | healthy | Burg Breuberg | 03.09.2019 | 49.827 | 9.02706 | hSouth |  |  |
| S_115 | damaged | Burg Breuberg | 03.09.2019 | 49.82689 | 9.02076 | dSouth |  |  |
| S_116 | healthy | Burg Breuberg | 03.09.2019 | 49.82689 | 9.02076 | hSouth |  |  |
| S_117 | damaged | Burg Breuberg | 03.09.2019 | 49.82692 | 9.02038 |  |  | x |
| S_118 | healthy | Burg Breuberg | 03.09.2019 | 49.82692 | 9.02038 |  |  | x |
| S_119 | damaged | Burg Breuberg | 03.09.2019 | 49.82322 | 9.02107 |  |  |  |
| S_120 | healthy | Burg Breuberg | 03.09.2019 | 49.82322 | 9.02107 |  |  |  |
| S_121 | damaged | Burg Breuberg | 03.09.2019 | 49.82305 | 9.02048 |  |  |  |
| S_122 | healthy | Burg Breuberg | 03.09.2019 | 49.82305 | 9.02048 |  |  |  |
| S_123 | damaged | Burg Breuberg | 03.09.2019 | 49.82301 | 9.02096 |  |  |  |
| S_124 | healthy | Burg Breuberg | 03.09.2019 | 49.82301 | 9.02096 |  |  |  |
| S_125 | damaged | Heusenstamm | 04.09.2019 | 50.04919 | 8.69445 | dSouth |  |  |
| S_126 | healthy | Heusenstamm | 04.09.2019 | 50.04919 | 8.69445 | hSouth |  |  |
| S_127 | damaged | Heusenstamm | 04.09.2019 | 50.04919 | 8.69445 | dSouth |  |  |
| S_128 | healthy | Heusenstamm | 04.09.2019 | 50.04919 | 8.69445 | hSouth |  |  |
| S_129 | damaged | Heusenstamm | 04.09.2019 | 50.0577 | 8.7788 |  |  | x |
| S_130 | healthy | Heusenstamm | 04.09.2019 | 50.0577 | 8.7788 |  |  | x |
| S_131 | damaged | Heusenstamm | 04.09.2019 | 50.0575 | 8.7787 | dSouth |  |  |
| S_132 | healthy | Heusenstamm | 04.09.2019 | 50.0575 | 8.7787 | hSouth |  |  |
| S_133 | damaged | Heusenstamm | 04.09.2019 | 50.05774 | 8.7796 | dSouth |  |  |
| S_134 | healthy | Heusenstamm | 04.09.2019 | 50.05774 | 8.7796 | hSouth |  |  |
| S_135 | damaged | Heusenstamm | 04.09.2019 | 50.05574 | 8.7795 | dSouth |  |  |
| S_136 | healthy | Heusenstamm | 04.09.2019 | 50.05574 | 8.7795 | hSouth |  |  |
| S_137 | damaged | Heusenstamm | 04.09.2019 | 50.0562 | 8.7787 | dSouth |  |  |
| S_138 | healthy | Heusenstamm | 04.09.2019 | 50.0562 | 8.7787 | hSouth |  |  |
| S_139 | damaged | Heusenstamm | 04.09.2019 | 50.0562 | 8.7787 | dSouth |  |  |
| S_140 | healthy | Heusenstamm | 04.09.2019 | 50.0562 | 8.7787 | hSouth |  |  |
| S_141 | damaged | Heusenstamm | 04.09.2019 | 50.0562 | 8.7787 | dSouth |  |  |
| S_142 | healthy | Heusenstamm | 04.09.2019 | 50.0562 | 8.7787 | hSouth |  |  |
| S_143 | damaged | Heusenstamm | 04.09.2019 | 50.0562 | 8.7787 |  |  | x |
| S_144 | healthy | Heusenstamm | 04.09.2019 | 50.0562 | 8.7787 |  |  | x |
| S_145 | damaged | Heusenstamm | 04.09.2019 | 50.0556 | 8.7788 |  |  | x |
| S_146 | healthy | Heusenstamm | 04.09.2019 | 50.0556 | 8.7788 |  |  | x |
| S_147 | damaged | Heusenstamm | 04.09.2019 | 50.056 | 8.78 |  |  |  |
| S_148 | healthy | Heusenstamm | 04.09.2019 | 50.056 | 8.78 |  |  |  |
| S_149 | damaged | Heusenstamm | 04.09.2019 | 50.0557 | 8.779 |  |  |  |
| S_150 | healthy | Heusenstamm | 04.09.2019 | 50.0557 | 8.779 |  |  |  |
| S_151 | damaged | Heusenstamm | 04.09.2019 | 50.0557 | 8.779 |  |  |  |
| S_152 | healthy | Heusenstamm | 04.09.2019 | 50.0557 | 8.779 |  |  |  |
| S_153 | damaged | Heusenstamm | 04.09.2019 | 50.0552 | 8.7797 |  |  |  |
| S_154 | healthy | Heusenstamm | 04.09.2019 | 50.0552 | 8.7797 |  |  |  |
| S_155 | damaged | Eschollbrücken | 06.09.2019 | 49.844862 | 8.6033987 | dSouth |  |  |
| S_156 | healthy | Eschollbrücken | 06.09.2019 | 49.844862 | 8.6033987 | hSouth |  |  |
| S_157 | damaged | Eschollbrücken | 06.09.2019 | 49.8451033 | 8.6033906 | dSouth |  |  |
| S_158 | healthy | Eschollbrücken | 06.09.2019 | 49.8451033 | 8.6033906 | hSouth |  |  |
| S_159 | damaged | Eschollbrücken | 06.09.2019 | 49.8452741 | 8.6933477 | dSouth |  |  |
| S_160 | healthy | Eschollbrücken | 06.09.2019 | 49.8452741 | 8.6933477 | hSouth |  |  |
| S_161 | damaged | Eschollbrücken | 06.09.2019 | 49.8464188 | 8.6058807 | dSouth |  |  |
| S_162 | healthy | Eschollbrücken | 06.09.2019 | 49.8464188 | 8.6058807 | hSouth |  |  |
| S_163 | damaged | Eschollbrücken | 06.09.2019 | 49.8461255 | 8.6064631 |  |  | x |
| S_164 | healthy | Eschollbrücken | 06.09.2019 | 49.8461255 | 8.6064631 |  |  | x |
| S_165 | damaged | Eschollbrücken | 06.09.2019 | 49.8381468 | 8.613406 |  |  |  |
| S_166 | healthy | Eschollbrücken | 06.09.2019 | 49.8381468 | 8.613406 |  |  |  |
| S_167 | damaged | Eschollbrücken | 06.09.2019 | 49.8371837 | 8.6154874 |  |  |  |
| S_168 | healthy | Eschollbrücken | 06.09.2019 | 49.8371837 | 8.6154874 |  |  |  |
| S_169 | damaged | Eschollbrücken | 06.09.2019 | 49.8368364 | 8.6172492 |  |  | x |
| S_170 | healthy | Eschollbrücken | 06.09.2019 | 49.8368364 | 8.6172492 |  |  | x |
| S_171 | damaged | Eschollbrücken | 06.09.2019 | 49.8364971 | 8.6193427 |  |  |  |
| S_172 | healthy | Eschollbrücken | 06.09.2019 | 49.8364971 | 8.6193427 |  |  |  |
| S_173 | damaged | Eschollbrücken | 06.09.2019 | 49.8366439 | 8.617927 |  |  |  |
| S_174 | healthy | Eschollbrücken | 06.09.2019 | 49.8366439 | 8.617927 |  |  |  |
| S_175 | damaged | Eschollbrücken | 06.09.2019 | 49.83549 | 8.6063276 |  |  | x |
| S_176 | healthy | Eschollbrücken | 06.09.2019 | 49.83549 | 8.6063276 |  |  | x |
| S_177 | damaged | Eschollbrücken | 06.09.2019 | 49.836002 | 8.6057516 |  |  |  |
| S_178 | healthy | Eschollbrücken | 06.09.2019 | 49.836002 | 8.6057516 |  |  |  |
| S_179 | damaged | Eschollbrücken | 06.09.2019 | 49.8364465 | 8.6051897 |  |  | x |
| S_180 | healthy | Eschollbrücken | 06.09.2019 | 49.8364465 | 8.6051897 |  |  | x |
| S_181 | damaged | Neu Isenburg | 09.09.2019 | 50.0643187 | 8.7090884 | dSouth |  |  |
| S_182 | healthy | Neu Isenburg | 09.09.2019 | 50.0643187 | 8.7090884 | hSouth |  |  |
| S_183 | damaged | Neu Isenburg | 09.09.2019 | 50.0650797 | 8.7098096 | dSouth |  |  |
| S_184 | healthy | Neu Isenburg | 09.09.2019 | 50.0650797 | 8.7098096 | hSouth |  |  |
| S_185 | damaged | Neu Isenburg | 09.09.2019 | 50.0659154 | 8.7102773 | dSouth |  |  |
| S_186 | healthy | Neu Isenburg | 09.09.2019 | 50.0659154 | 8.7102773 | hSouth |  |  |
| S_187 | damaged | Neu Isenburg | 09.09.2019 | 50.0659301 | 8.7100359 | dSouth |  |  |
| S_188 | healthy | Neu Isenburg | 09.09.2019 | 50.0659301 | 8.7100359 | hSouth |  |  |
| S_189 | damaged | Neu Isenburg | 09.09.2019 | 50.0686111 | 8.7121451 | dSouth |  |  |
| S_190 | healthy | Neu Isenburg | 09.09.2019 | 50.0686111 | 8.7121451 | hSouth |  |  |
| S_191 | damaged | Neu Isenburg | 09.09.2019 | 50.0691756 | 8.7123064 | dSouth |  |  |
| S_192 | healthy | Neu Isenburg | 09.09.2019 | 50.0691756 | 8.7123064 | hSouth |  |  |
| S_193 | damaged | Neu Isenburg | 09.09.2019 | 50.0710306 | 8.7127781 | dSouth |  |  |
| S_194 | healthy | Neu Isenburg | 09.09.2019 | 50.0710306 | 8.7127781 | hSouth |  |  |
| S_195 | damaged | Neu Isenburg | 09.09.2019 | 50.0716691 | 8.7111252 | dSouth |  |  |
| S_196 | healthy | Neu Isenburg | 09.09.2019 | 50.0716691 | 8.7111252 | hSouth |  |  |
| S_197 | damaged | Neu Isenburg | 09.09.2019 | 50.0715402 | 8.7110297 |  |  |  |
| S_198 | healthy | Neu Isenburg | 09.09.2019 | 50.0715402 | 8.7110297 |  |  |  |
| S_199 | damaged | Neu Isenburg | 09.09.2019 | 50.0710603 | 8.7121176 |  |  |  |
| S_200 | healthy | Neu Isenburg | 09.09.2019 | 50.0710603 | 8.7121176 |  |  |  |
| S_201 | damaged | Neu Isenburg | 09.09.2019 | 50.0681943 | 8.7052592 |  |  |  |
| S_202 | healthy | Neu Isenburg | 09.09.2019 | 50.0681943 | 8.7052592 |  |  |  |
| S_203 | damaged | Neu Isenburg | 09.09.2019 | 50.0671452 | 8.7042427 |  |  |  |
| S_204 | healthy | Neu Isenburg | 09.09.2019 | 50.0671452 | 8.7042427 |  |  |  |
| S_205 | damaged | Wegscheide | 10.09.2019 | 50.2076 | 9.4126 |  |  | x |
| S_206 | healthy | Wegscheide | 10.09.2019 | 50.2076 | 9.4126 |  |  | x |
| S_207 | damaged | Wegscheide | 10.09.2019 | 50.2076 | 9.4126 |  |  |  |
| S_208 | healthy | Wegscheide | 10.09.2019 | 50.2076 | 9.4126 |  |  |  |
| S_209 | damaged | Wegscheide | 10.09.2019 | 50.2149354 | 9.419513 |  |  |  |
| S_210 | healthy | Wegscheide | 10.09.2019 | 50.2149354 | 9.419513 |  |  |  |
| S_211 | damaged | Wegscheide | 10.09.2019 | 50.2114485 | 9.409662 |  |  |  |
| S_212 | healthy | Wegscheide | 10.09.2019 | 50.2114485 | 9.409662 |  |  |  |
| S_213 | damaged | Wegscheide | 10.09.2019 | 50.2114485 | 9.409662 | dSouth |  |  |
| S_214 | healthy | Wegscheide | 10.09.2019 | 50.2114485 | 9.409662 | hSouth |  |  |
| S_215 | damaged | Wegscheide | 10.09.2019 | 50.2109 | 9.4086 | dSouth |  |  |
| S_216 | healthy | Wegscheide | 10.09.2019 | 50.2109 | 9.4086 | hSouth |  |  |
| S_217 | damaged | Waldstadion | 10.09.2019 | 50.0546504 | 8.6626215 | dSouth |  |  |
| S_218 | healthy | Waldstadion | 10.09.2019 | 50.0546504 | 8.6626215 | hSouth |  |  |
| S_219 | damaged | Waldstadion | 10.09.2019 | 50.0555269 | 8.6618698 | dSouth |  |  |
| S_220 | healthy | Waldstadion | 10.09.2019 | 50.0555269 | 8.6618698 | hSouth |  |  |
| S_221 | damaged | Waldstadion | 10.09.2019 | 50.0568445 | 8.6610993 | dSouth |  |  |
| S_222 | healthy | Waldstadion | 10.09.2019 | 50.0568445 | 8.6610993 | hSouth |  |  |
| S_223 | damaged | Waldstadion | 10.09.2019 | 50.0572346 | 8.6619006 | dSouth |  |  |
| S_224 | healthy | Waldstadion | 10.09.2019 | 50.0572346 | 8.6619006 | hSouth |  |  |
| S_225 | damaged | Waldstadion | 10.09.2019 | 50.0573499 | 8.661843 | dSouth |  |  |
| S_226 | healthy | Waldstadion | 10.09.2019 | 50.0573499 | 8.661843 | hSouth |  |  |
| S_227 | damaged | Waldstadion | 10.09.2019 | 50.0589663 | 8.6609092 | dSouth |  |  |
| S_228 | healthy | Waldstadion | 10.09.2019 | 50.0589663 | 8.6609092 | hSouth |  |  |
| S_229 | damaged | Waldstadion | 10.09.2019 | 50.0587827 | 8.6609424 | dSouth |  |  |
| S_230 | healthy | Waldstadion | 10.09.2019 | 50.0587827 | 8.6609424 | hSouth |  |  |
| S_231 | damaged | Messel | 11.09.2019 | 49.9648055 | 8.7305273 | dSouth |  |  |
| S_232 | healthy | Messel | 11.09.2019 | 49.9648055 | 8.7305273 | hSouth |  |  |
| S_233 | damaged | Messel | 11.09.2019 | 49.9653231 | 8.7286833 | dSouth |  |  |
| S_234 | healthy | Messel | 11.09.2019 | 49.9653231 | 8.7286833 | hSouth |  |  |
| S_235 | damaged | Messel | 11.09.2019 | 49.9672891 | 8.7267089 | dSouth |  |  |
| S_236 | healthy | Messel | 11.09.2019 | 49.9672891 | 8.7267089 | hSouth |  |  |
| S_237 | damaged | Messel | 11.09.2019 | 49.9673648 | 8.726613 | dSouth |  |  |
| S_238 | healthy | Messel | 11.09.2019 | 49.9673648 | 8.726613 | hSouth |  |  |
| S_239 | damaged | Messel | 11.09.2019 | 49.9685641 | 8.7259826 | dSouth |  |  |
| S_240 | healthy | Messel | 11.09.2019 | 49.9685641 | 8.7259826 | hSouth |  |  |
| S_241 | damaged | Messel | 11.09.2019 | 49.9702459 | 8.7244602 | dSouth |  |  |
| S_242 | healthy | Messel | 11.09.2019 | 49.9702459 | 8.7244602 | hSouth |  |  |
| S_243 | damaged | Messel | 11.09.2019 | 49.9722449 | 8.7156846 | dSouth |  |  |
| S_244 | healthy | Messel | 11.09.2019 | 49.9722449 | 8.7156846 | hSouth |  |  |
| S_245 | damaged | Messel | 11.09.2019 | 49.9722449 | 8.7156806 |  |  | x |
| S_246 | healthy | Messel | 11.09.2019 | 49.9722449 | 8.7156806 |  |  | x |
| S_247 | damaged | Messel | 11.09.2019 | 49.9724476 | 8.7142302 | dSouth |  |  |
| S_248 | healthy | Messel | 11.09.2019 | 49.9724476 | 8.7142302 | hSouth |  |  |
| S_249 | damaged | Messel | 11.09.2019 | 49.9718971 | 8.7119104 | dSouth |  |  |
| S_250 | healthy | Messel | 11.09.2019 | 49.9718971 | 8.7119104 | hSouth |  |  |
| S_251 | damaged | Messel | 11.09.2019 | 49.9719081 | 8.7114799 | dSouth |  |  |
| S_252 | healthy | Messel | 11.09.2019 | 49.9719081 | 8.7114799 | hSouth |  |  |
| S_253 | damaged | Lorsch | 12.09.2019 | 49.6491886 | 8.5376088 | dSouth |  |  |
| S_254 | healthy | Lorsch | 12.09.2019 | 49.6491886 | 8.5376088 | hSouth |  |  |
| S_255 | damaged | Lorsch | 12.09.2019 | 49.6493532 | 8.5369721 | dSouth |  |  |
| S_256 | healthy | Lorsch | 12.09.2019 | 49.6493532 | 8.5369721 | hSouth |  |  |
| S_257 | damaged | Lorsch | 12.09.2019 | 49.6488428 | 8.5381241 | dSouth |  |  |
| S_258 | healthy | Lorsch | 12.09.2019 | 49.6488428 | 8.5381241 | hSouth |  |  |
| S_259 | damaged | Lorsch | 12.09.2019 | 49.6485083 | 8.5392349 | dSouth |  |  |
| S_260 | healthy | Lorsch | 12.09.2019 | 49.6485083 | 8.5392349 | hSouth |  |  |
| S_261 | damaged | Lorsch | 12.09.2019 | 49.6481419 | 8.5395785 | dSouth |  |  |
| S_262 | healthy | Lorsch | 12.09.2019 | 49.6481419 | 8.5395785 | hSouth |  |  |
| S_263 | damaged | Lorsch | 12.09.2019 | 49.6462656 | 8.5421035 | dSouth |  |  |
| S_264 | healthy | Lorsch | 12.09.2019 | 49.6462656 | 8.5421035 | hSouth |  |  |
| S_265 | damaged | Lorsch | 12.09.2019 | 49.6464364 | 8.5415305 |  |  | x |
| S_266 | healthy | Lorsch | 12.09.2019 | 49.6464364 | 8.5415305 |  |  | x |
| S_267 | damaged | Lorsch | 12.09.2019 | 49.6463205 | 8.5410504 |  |  |  |
| S_268 | healthy | Lorsch | 12.09.2019 | 49.6463205 | 8.5410504 |  |  |  |
| S_269 | damaged | Lorsch | 12.09.2019 | 49.6452786 | 8.5358174 |  |  |  |
| S_270 | healthy | Lorsch | 12.09.2019 | 49.6452786 | 8.5358174 |  |  |  |
| S_271 | damaged | Lorsch | 12.09.2019 | 49.6451675 | 8.5351421 |  |  |  |
| S_272 | healthy | Lorsch | 12.09.2019 | 49.6451675 | 8.5351421 |  |  |  |
| S_273 | damaged | Lorsch | 12.09.2019 | 49.649297 | 8.5362281 |  |  | x |
| S_274 | healthy | Lorsch | 12.09.2019 | 49.649297 | 8.5362281 |  |  | x |
| S_275 | damaged | Lorsch | 12.09.2019 | 49.6494678 | 8.5366794 |  |  |  |
| S_276 | healthy | Lorsch | 12.09.2019 | 49.6494678 | 8.5366794 |  |  |  |
| S_277 | damaged | Heusenstamm | 13.09.2019 | 50.0580508 | 8.7747529 |  |  |  |
| S_278 | healthy | Heusenstamm | 13.09.2019 | 50.0580508 | 8.7747529 |  |  |  |
| S_279 | damaged | Heusenstamm | 13.09.2019 | 50.0580635 | 8.7746208 |  |  | x |
| S_280 | healthy | Heusenstamm | 13.09.2019 | 50.0580635 | 8.7746208 |  |  | x |
| S_281 | damaged | Heusenstamm | 13.09.2019 | 50.057182 | 8.773846 | dSouth |  |  |
| S_282 | healthy | Heusenstamm | 13.09.2019 | 50.057182 | 8.773846 | hSouth |  |  |
| S_283 | damaged | Heusenstamm | 13.09.2019 | 50.0571046 | 8.7740264 | dSouth |  |  |
| S_284 | healthy | Heusenstamm | 13.09.2019 | 50.0571046 | 8.7740264 | hSouth |  |  |
| S_285 | damaged | Heusenstamm | 13.09.2019 | 50.0566422 | 8.7736616 | dSouth |  |  |
| S_286 | healthy | Heusenstamm | 13.09.2019 | 50.0566422 | 8.7736616 | hSouth |  |  |
| S_287 | damaged | Heusenstamm | 13.09.2019 | 50.0561296 | 8.7732438 | dSouth |  |  |
| S_288 | healthy | Heusenstamm | 13.09.2019 | 50.0561296 | 8.7732438 | hSouth |  |  |
| S_289 | damaged | Heusenstamm | 13.09.2019 | 50.0559955 | 8.7728465 | dSouth |  |  |
| S_290 | healthy | Heusenstamm | 13.09.2019 | 50.0559955 | 8.7728465 | hSouth |  |  |
| S_291 | damaged | Heusenstamm | 13.09.2019 | 50.055472 | 8.7729314 | dSouth |  |  |
| S_292 | healthy | Heusenstamm | 13.09.2019 | 50.055472 | 8.7729314 | hSouth |  |  |
| S_293 | damaged | Heusenstamm | 13.09.2019 | 50.055181 | 8.772228 | dSouth |  |  |
| S_294 | healthy | Heusenstamm | 13.09.2019 | 50.055181 | 8.772228 | hSouth |  |  |
| S_295 | damaged | Heusenstamm | 13.09.2019 | 50.0551293 | 8.7723349 | dSouth |  |  |
| S_296 | healthy | Heusenstamm | 13.09.2019 | 50.0551293 | 8.7723349 | hSouth |  |  |
| S_297 | damaged | Heusenstamm | 13.09.2019 | 50.0549188 | 8.7721502 | dSouth |  |  |
| S_298 | healthy | Heusenstamm | 13.09.2019 | 50.0549188 | 8.7721502 | hSouth |  |  |
| S_299 | damaged | Heusenstamm | 13.09.2019 | 50.0548086 | 8.7726598 |  |  | X |
| S_300 | healthy | Heusenstamm | 13.09.2019 | 50.0548086 | 8.7726598 |  |  | X |
| conf_001 | damaged | Schloßborn | 03.08.2020 | 50.1904623 | 8.373935 |  |  | x |
| conf_002 | healthy | Schloßborn | 03.08.2020 | 50.1902617 | 8.3738026 |  |  | X |
| conf_003 | damaged | Schloßborn | 03.08.2020 | 50.1887401 | 8.372701 |  |  |  |
| conf_004 | healthy | Schloßborn | 03.08.2020 | 50.1879031 | 8.3716613 |  |  | X |
| conf_005 | damaged | Schloßborn | 03.08.2020 | 50.1865722 | 8.3710684 |  |  | X |
| conf_006 | healthy | Schloßborn | 03.08.2020 | 50.18656127 | 8.3711265 |  |  | X |
| conf_007 | damaged | Schloßborn | 03.08.2020 | 50.1857023 | 8.3708097 |  |  | X |
| conf_008 | healthy | Schloßborn | 03.08.2020 | 50.1851555 | 8.3712359 |  |  | X |
| conf_009 | damaged | Schloßborn | 03.08.2020 | 50.1852938 | 8.371852 |  |  | X |
| conf_010 | healthy | Schloßborn | 03.08.2020 | 50.1851807 | 8.3722412 |  |  | X |
| conf_011 | healthy | Schloßborn | 03.08.2020 | 50.1864421 | 8.3767229 |  |  | X |
| conf_012 | damaged | Schloßborn | 03.08.2020 | 50.1864287 | 8.3764222 |  |  | X |
| conf_013 | damaged | Ruppertshain | 04.08.2020 | 50.181629 | 8.4018675 |  |  | X |
| conf_014 | healthy | Ruppertshain | 04.08.2020 | 50.1815555 | 8.4018799 |  |  | X |
| conf_016 | healthy | Ruppertshain | 04.08.2020 | 50.1818417 | 8.4023975 |  |  | X |
| conf_017 | damaged | Ruppertshain | 04.08.2020 | 50.1846569 | 8.4022888 |  |  | X |
| conf_018 | healthy | Ruppertshain | 04.08.2020 | 50.1847183 | 8.40225810 |  |  | X |
| conf_019 | damaged | Ruppertshain | 04.08.2020 | 50.1867665 | 8.4029614 |  |  | X |
| conf_020 | healthy | Ruppertshain | 04.08.2020 | 50.1868466 | 8.4029997 |  |  | X |
| conf_021 | damaged | Ruppertshain | 04.08.2020 | 50.1868411 | 8.4040772 |  |  | X |
| conf_022 | healthy | Ruppertshain | 04.08.2020 | 50.1877796 | 8.4046797 |  |  | X |
| conf_023 | damaged | Ruppertshain | 04.08.2020 | 50.1829212 | 8.3987642 |  |  | X |
| conf_024 | healthy | Ruppertshain | 04.08.2020 | 50.182956 | 8.3987399 |  |  | X |
| conf_025 | damaged | Ruppertshain | 04.08.2020 | 50.1808246 | 8.3990728 |  |  | X |
| conf_026 | healthy | Ruppertshain | 04.08.2020 | 50.1806971 | 8.39899759 |  |  | X |
| conf_027 | damaged | Ruppertshain | 04.08.2020 | 50.179967 | 8.3994577 |  |  | X |
| conf_028 | healthy | Ruppertshain | 04.08.2020 | 50.1798082 | 8.399525 |  |  | X |
| conf_029 | damaged | Ruppertshain | 04.08.2020 | 50.1782612 | 8.3999872 |  |  | X |
| conf_030 | healthy | Ruppertshain | 04.08.2020 | 50.1782916 | 8.4002846 |  |  | X |
| conf_032 | healthy | Falkenstein | 05.08.2020 | 50.201454 | 8.4872785 |  |  | X |
| conf_033 | damaged | Falkenstein | 05.08.2020 | 50.2015038 | 8.4874409 |  |  | X |
| conf_034 | damaged | Falkenstein | 05.08.2020 | 50.201115 | 8.4876516 |  |  | X |
| conf_036 | healthy | Falkenstein | 05.08.2020 | 50.2018627 | 8.4875841 |  |  | X |
| conf_037 | damaged | Falkenstein | 05.08.2020 | 50.2011419 | 8.4892266 |  |  | x |
| conf_038 | healthy | Falkenstein | 05.08.2020 | 50.2012289 | 8.489425 |  |  | x |
| conf_039 | healthy | Falkenstein | 05.08.2020 | 50.2013307 | 8.4905266 |  |  |  |
| conf_040 | damaged | Falkenstein | 05.08.2020 | 50.2004517 | 8.4905881 |  |  | x |
| conf_041 | healthy | Falkenstein | 05.08.2020 | 50.2008376 | 8.4909408 |  |  | x |
| conf_042 | damaged | Falkenstein | 05.08.2020 | 50.20009229 | 8.4909295 |  |  | x |
| conf_043 | damaged | Falkenstein | 05.08.2020 | 50.1997086 | 8.4828883 |  |  | x |
| conf_045 | healthy | Falkenstein | 05.08.2020 | 50.1992717 | 8.480502 |  |  | x |
| conf_046 | damaged | Falkenstein | 05.08.2020 | 50.1995271 | 8.4806157 |  |  | x |
| conf_047 | healthy | Falkenstein | 05.08.2020 | 50.1995032 | 8.4806215 |  |  | x |
| conf_050 | damaged | Rote Mühle | 07.08.2020 | 50.1612598 | 8.452304 |  |  | x |
| conf_051 | healthy | Rote Mühle | 07.08.2020 | 50.1610649 | 8.4526065 |  |  | x |
| conf_052 | damaged | Rote Mühle | 07.08.2020 | 50.1611651 | 8.4520887 |  |  | x |
| conf_053 | healthy | Rote Mühle | 07.08.2020 | 50.1611113 | 8.4520672 |  |  | x |
| conf_054 | damaged | Rote Mühle | 07.08.2020 | 50.1611776 | 8.4511498 |  |  | x |
| conf_055 | healthy | Rote Mühle | 07.08.2020 | 50.1611331 | 8.450964 |  |  | x |
| conf_056 | damaged | Rote Mühle | 07.08.2020 | 50.1612598 | 8.4508452 |  |  | x |
| conf_057 | healthy | Rote Mühle | 07.08.2020 | 50.1614085 | 8.4509926 |  |  | x |
| conf_058 | damaged | Rote Mühle | 07.08.2020 | 50.1617899 | 8.4504762 |  |  | x |
| conf_059 | healthy | Rote Mühle | 07.08.2020 | 50.1617076 | 8.4505298 |  |  | x |

**Spplementary file 1B**. Results from Mann-Whitney U-test on difference between damaged and healthy trees for various parameters.

| Parameter | Mann-Whitney U | p |
| --- | --- | --- |
| trunk circumference | 10718 | 0.48 |
| tree height | 9874 | 0.27 |
| canopy closure | 10820 | 0.56 |
| competition index | 10809 | 0.57 |
| dried leaves | 202 | **<0.001** |
| leaf loss | 352 | **<0.001** |

**Supplementary file 1C**. List of genes with significant SNPs. Functional annotations and relation to drought phenotype are given whenever available.

| ***F. sylvatica* gene ID** | **Best BLAST hit ID** | **BLAST hit protein name** | **taxon** | **UniProt ID** | **UniProt protein name** | **function (UniProt)** | **citation function** | **relation to drought phenotype** | **citation** |
| --- | --- | --- | --- | --- | --- | --- | --- | --- | --- |
| 1.g3851.t1 | KUM50718.1 | hypothetical protein ABT39_MTgene562 | *Picea glauca* | none | - | - | - | - | - |
| 10.g3914.t1 | XP_023883481.1 | uncharacterized protein LOC111995782 isoform X1 | *Quercus suber* | none | - | - | - | - | - |
| 11.g2467.t1 | XP_023923514.1 | exosome complex exonuclease RRP46 homolog isoform X1 | *Quercus suber* | EXOS5_ORYSJ | Exosome complex exonuclease RRP46 homolog | mRNA degradation | Xiang, D., Yang, H., Venglat, P., Cao, Y., Wen, R., Ren, M., ... & Weijers, D. (2011). POPCORN functions in the auxin pathway to regulate embryonic body plan and meristem organization in Arabidopsis. The Plant Cell, 23(12), 4348-4367. | - | - |
| 11.g2832.t1 | XP_030949821.1 | WD repeat-containing protein PCN-like | *Quercus lobata* | PCN_ARATH | WD repeat-containing protein PCN | Involved in auxin signalling pathway. Required for embryo development and meristem organization. Functions in the auxin pathway, integrating auxin signalling in the organization and maintenance of the shoot apical meristem (SAM) and root apical meristem (RAM). | - | drought stress response | Park, S. R., Hwang, J., & Kim, M. (2020). The Arabidopsis WDR55 is positively involved in ABA-mediated drought tolerance response. Plant Biotechnology Reports, 1-12. |
| 12.g1695.t1 | XP_030973623.1 | uncharacterized protein LOC115993791 | *Quercus lobata* | F4I5S1_ARATH | PB1 domain-containing protein tyrosine kinase | not well characterised | - | - | - |
| 2.g4736.t1 | KAF3975221.1 | hypothetical protein CMV_001513 | *Castanea mollissima* | none | - | - | - | - | - |
| 3.g3590.t1 | XP_030942104.1 | uncharacterized protein LOC115967180 | *Quercus lobata* | none | - | - | - | - | - |
| 4.g3980.t1 | XP_030950630.1 | cytokinin dehydrogenase 5 | *Quercus lobata* | CKX1_ARATH | Cytokinin dehydrogenase 1 | Catalyzes the oxidation of cytokinins, a family of N6-substituted adenine derivatives that are plant hormones, where the substituent is an isopentenyl group. | Werner, T., Motyka, V., Laucou, V., Smets, R., Van Onckelen, H., & Schmülling, T. (2003). Cytokinin-deficient transgenic Arabidopsis plants show multiple developmental alterations indicating opposite functions of cytokinins in the regulation of shoot and root meristem activity. The Plant Cell, 15(11), 2532-2550. | drought stress response | Emery, R. J., & Kisiala, A. (2020). The Roles of Cytokinins in Plants and Their Response to Environmental Stimuli. Li, W., Herrera-Estrella, L., & Tran, L. S. P. (2016). The Yin–Yang of cytokinin homeostasis and drought acclimation/adaptation. Trends in Plant Science, 21(7), 548-550. |
| 5.g1807.t1 | XP_030939294.1 | guanosine nucleotide diphosphate dissociation inhibitor 2 | *Quercus lobata* | GDI2_ARATH | Guanosine nucleotide diphosphate dissociation inhibitor 2 | Regulates the GDP/GTP exchange reaction of most RAB proteins by inhibiting the dissociation of GDP from them, and the subsequent binding of GTP. | Ueda, T., Yoshizumi, T., Anai, T., Matsui, M., Uchimiya, H., & Nakano, A. (1998). AtGDI2, a novel Arabidopsis gene encoding a Rab GDP dissociation inhibitor. Gene, 206(1), 137-143. | environmental stress response | Carvalho, B. M., Viana, A. P., dos Santos, P. H. D., Generoso, A. L., Corrêa, C. C. G., Silveira, V., ... & Santos, E. A. (2019). Proteome of resistant and susceptible Passiflora species in the interaction with cowpea aphid-borne mosaic virus reveals distinct responses to pathogenesis. Euphytica, 215(10), 167. |
| 6.g2227.t1 | THG21676.1 | hypothetical protein TEA_000305 | *Camellia sinensis* var. *sinensis* | NDUS7_ARATH | NADH dehydrogenase [ubiquinone] iron-sulfur protein 7, mitochondrial | Core subunit of the mitochondrial membrane respiratory chain NADH dehydrogenase (Complex I) that is believed to belong to the minimal assembly required for catalysis. Complex I functions in the transfer of electrons from NADH to the respiratory chain. The immediate electron acceptor for the enzyme is believed to be ubiquinone |  | drought stress response | Zhang, S., Zhang, L., Zhou, K., Li, Y., & Zhao, Z. (2017). Changes in protein profile of Platycladus orientalis (L.) roots and leaves in response to drought stress. Tree Genetics & Genomes, 13(4), 76. |
| 6.g2921.t1 | KAB1222126.1 | Ribonuclease H2 subunit C | *Morella rubra* | RNH2A_ARATH | Ribonuclease H2 subunit A | Catalytic subunit of RNase HII, an endonuclease that specifically degrades the RNA of RNA:DNA hybrids. Participates in DNA replication, possibly by mediating the removal of lagging-strand Okazaki fragment RNA primers during DNA replication. Mediates the excision of single ribonucleotides from DNA:RNA duplexes | - | - | - |
| 7.g177.t1 | XP_018842121.1 | PREDICTED: tubby-like F-box protein 8 isoform X1 | *Juglans regia* | TLP10_ARATH | Tubby-like F-box protein 10 | Component of SCF(ASK-cullin-F-box) E3 ubiquitin ligase complexes, which may mediate the ubiquitination and subsequent proteasomal degradation of target proteins | - | drought stress response | Xu, J., Xing, S., Sun, Q., Zhan, C., Liu, X., Zhang, S., & Wang, X. (2019). The expression of a tubby-like protein from Malus domestica (Md TLP7) enhances abiotic stress tolerance in Arabidopsis. BMC plant biology, 19(1), 1-8. |
| 7.g1655.t1 | XP_023909357.1 | histone deacetylase 6 | *Quercus suber* | HDA6 | Histone deacetylase 6 | Responsible for the deacetylation of lysine residues on the N-terminal part of the core histones (H2A, H2B, H3 and H4). Histone deacetylation gives a tag for epigenetic repression and plays an important role in transcriptional regulation, cell cycle progression and developmental events. | "Identification of Arabidopsis histone deacetylase HDA6 mutants that affect transgene expression."  Murfett J., Wang X.-J., Hagen G., Guilfoyle T.J.  Plant Cell 13:1047-1061(2001) | drought stress | Zheng, Y., Ding, Y., Sun, X., Xie, S., Wang, D., Liu, X., ... & Zhou, D. X. (2016). Histone deacetylase HDA9 negatively regulates salt and drought stress responsiveness in Arabidopsis. Journal of experimental botany, 67(6), 1703-1713. |
| 7.g2350.t1 | KAF3966828.1 | hypothetical protein CMV_009102 | *Castanea mollissima* | PRK4_ARATH | Pollen receptor-like kinase 4 | Receptor-like kinase involved in the control of pollen germination and pollen tube polar growth. Can phosphorylate ROPGEF1 in vitro | Chang, F., Gu, Y., Ma, H., & Yang, Z. (2013). AtPRK2 promotes ROP1 activation via RopGEFs in the control of polarized pollen tube growth. Molecular plant, 6(4), 1187-1201. | environmental stress response | Guo, J., Dong, X., Li, Y., & Wang, B. (2020). NaCl treatment markedly enhanced pollen viability and pollen preservation time of euhalophyte Suaeda salsa via up regulation of pollen development-related genes. Journal of plant research, 133(1), 57-71. |
| 7.g3617.t1 | XP_023924613.1 | protein LIGHT-DEPENDENT SHORT HYPOCOTYLS 10-like isoform X1 | *Quercus suber* | LSH4_ARATH | Protein LIGHT-DEPENDENT SHORT HYPOCOTYLS 4 | Probable transcription regulator that acts as a developmental regulator by promoting cell growth in response to light. May suppress organ differentiation in the boundary region | Takeda, S., Hanano, K., Kariya, A., Shimizu, S., Zhao, L., Matsui, M., ... & Aida, M. (2011). CUP‐SHAPED COTYLEDON1 transcription factor activates the expression of LSH4 and LSH3, two members of the ALOG gene family, in shoot organ boundary cells. The Plant Journal, 66(6), 1066-1077. | - |  |
| 7.g3816.t1 | XP_018811587.1 | ethylene-responsive transcription factor 12-like | *Juglans regia* | TAFCL_ARATH | Transcription initiation factor TFIID subunit 12b | TAFs are components of the transcription factor IID (TFIID) complex that is essential for mediating regulation of RNA polymerase transcription. Required for the expression of a subset of ethylene-responsive genes (By similarity). Involved in the negative regulation of cytokinin sensitivity | - | - | - |
| 7.g552.t1 | No hits found |  | *-* | - | - | - | - | - | - |
| 8.g3494.t1 | XP_022750437.1 | V-type proton ATPase subunit C | *Durio zibethinus* | VATC_ARATH | V-type proton ATPase subunit C | Subunit of the peripheral V1 complex of vacuolar ATPase. Subunit C is necessary for the assembly of the catalytic sector of the enzyme and is likely to have a specific function in its catalytic activity. V-ATPase is responsible for acidifying a variety of intracellular compartments in eukaryotic cells | - | drought stress response | Kausar, R., Arshad, M., Shahzad, A., & Komatsu, S. (2013). Proteomics analysis of sensitive and tolerant barley genotypes under drought stress. Amino Acids, 44(2), 345-359. Li, J., Jia, H., Han, X., Zhang, J., Sun, P., Lu, M., & Hu, J. (2016). Selection of reliable reference genes for gene expression analysis under abiotic stresses in the desert biomass willow, *Salix psammophila*. Frontiers in plant science, 7, 1505. |
| 9.g3080.t1 | XP_030929046.1 | probable inactive purple acid phosphatase 29 | *Quercus lobata* | PPA14_ARATH | Probable inactive purple acid phosphatase 14 | - | - | drought stress response | Street, N. R., Skogström, O., Sjödin, A., Tucker, J., Rodríguez‐Acosta, M., Nilsson, P., ... & Taylor, G. (2006). The genetics and genomics of the drought response in Populus. The Plant Journal, 48(3), 321-341. Prinsi, B., Negri, A. S., Failla, O., Scienza, A., & Espen, L. (2018). Root proteomic and metabolic analyses reveal specific responses to drought stress in differently tolerant grapevine rootstocks. BMC plant biology, 18(1), 126. |
| 9.g4504.t1 | KAF3976808.1 | hypothetical protein CMV_000062 | *Castanea mollissima* | TBL33_ARATH | Protein trichome birefringence-like 33 | Probable xylan acetyltransferase that plays a role in xylan acetylation and normal deposition of secondary cell walls | Yuan, Y., Teng, Q., Zhong, R., Haghighat, M., Richardson, E. A., & Ye, Z. H. (2016). Mutations of Arabidopsis TBL32 and TBL33 affect xylan acetylation and secondary wall deposition. PLoS One, 11(1), e0146460. | drought stress response | Shuai, P., Liang, D., Zhang, Z., Yin, W., & Xia, X. (2013). Identification of drought-responsive and novel Populus trichocarpamicroRNAs by high-throughput sequencing and their targets using degradome analysis. Bmc Genomics, 14(1), 233. |

**Supplementary file 1D**. List of genes closest to significant SNPs. Functional annotations and relation to drought phenotype are given whenever available.

| ***F. sylvatica* gene ID** | **Best BLAST hit ID** | **BLAST hit protein name** | **taxon** | **UniProt ID** | **UniProt protein name** | **function (UniProt)** | **citation function** | **relation to drought phenotype** | **citation** |
| --- | --- | --- | --- | --- | --- | --- | --- | --- | --- |
| Backbone_621.g33 | KAF3957547 | hypothetical protein CMV_017449 | *Castanea mollissima* |  |  |  |  |  |  |
| Backbone_621.g49 | XP_030932040 | pathogen-related protein | *Quercus lobata* |  |  |  |  |  |  |
| 1.g1111 | KAF3976566 | hypothetical protein CMV_000247 | *Castanea mollissima* | ACBP4_ARATH | Acyl-CoA-binding domain-containing protein 4 | Binds medium- and long-chain acyl-CoA esters with very high affinity. Can interact in vitro with oleoyl-CoA, barely with palmitoyl-CoA, but not with arachidonyl-CoA. | Leung, K. C., Li, H. Y., Mishra, G., & Chye, M. L. (2005). ACBP4 and ACBP5, novel Arabidopsis acyl-CoA-binding proteins with kelch motifs that bind oleoyl-CoA. Plant molecular biology, 55(2), 297-309. | environmental stress response | Du, Z. Y., Arias, T., Meng, W., & Chye, M. L. (2016). Plant acyl-CoA-binding proteins: an emerging family involved in plant development and stress responses. Progress in Lipid Research, 63, 165-181. |
| 1.g2051 | XP_031256586 | aspartic proteinase nepenthesin-1-like | *Pistacia vera* |  |  |  |  | drought stress response | Prinsi, B., Negri, A. S., Failla, O., Scienza, A., & Espen, L. (2018). Root proteomic and metabolic analyses reveal specific responses to drought stress in differently tolerant grapevine rootstocks. BMC plant biology, 18(1), 126. |
| 1.g5025 | No hits found |  |  |  |  |  |  |  |  |
| 1.g6250 | XP_030925267 | BTB/POZ domain and ankyrin repeat-containing protein NOOT2 | *Quercus lobata* | NPR5_ARATH | Regulatory protein NPR5 | May act as a substrate-specific adapter of an E3 ubiquitin-protein ligase complex (CUL3-RBX1-BTB) which mediates the ubiquitination and subsequent proteasomal degradation of target proteins | Hepworth, S. R., Zhang, Y., McKim, S., Li, X., & Haughn, G. W. (2005). BLADE-ON-PETIOLE–dependent signaling controls leaf and floral patterning in Arabidopsis. The Plant Cell, 17(5), 1434-1448. |  |  |
| 1.g7050 | PQQ17864 | insulin-degrading enzyme-like 1 peroxisomal | *Prunus yedoensis* | IDE1_ARATH | insulin-degrading enzyme-like 1 peroxisomal | Peptidase that might be involved in pathogen or wound response. | Lingard, M. J., & Bartel, B. (2009). Arabidopsis LON2 is necessary for peroxisomal function and sustained matrix protein import. Plant physiology, 151(3), 1354-1365. |  |  |
| 2.g1138 | XP_018818502 | PREDICTED: uncharacterized protein LOC108989373 isoform X3 | *Juglans regia* |  |  |  |  |  |  |
| 2.g1372 | KAF3967401 | hypothetical protein CMV_008608 | *Castanea mollissima* |  |  |  |  |  |  |
| 2.g1982 | No hits found |  |  |  |  |  |  |  |  |
| 2.g4924 | TKY55186 | Spermine synthase | *Spatholobus suberectus* | SPSY_ARATH | Spermine synthase |  |  | drought stress response | Yamaguchi, K., Takahashi, Y., Berberich, T., Imai, A., Takahashi, T., Michael, A. J., & Kusano, T. (2007). A protective role for the polyamine spermine against drought stress in Arabidopsis. Biochemical and biophysical research communications, 352(2), 486-490. |
| 3.g1427 | XP_030940223 | uncharacterized protein LOC115965177 | *Quercus lobata* |  |  |  |  |  |  |
| 3.g4686 | KAF3964003 | hypothetical protein CMV_011671 | *Castanea mollissima* |  |  |  |  |  |  |
| 4.g1310 | KAF3954505 | hypothetical protein CMV_020159 | *Castanea mollissima* | Q9C6S8_ARATH | Pre-mRNA-splicing factor of RES complex protein |  |  | drought stress response | Qi, Y., Yao, X., Zhao, D., & Lu, L. (2018). Overexpression of SbSKIP, a pre-mRNA splicing factor from Sorghum bicolor, enhances root growth and drought tolerance in Petunia hybrida. Scientia Horticulturae, 240, 281-287. |
| 4.g3868 | KAF3971069 | hypothetical protein CMV_005309 | *Castanea mollissima* |  |  |  |  |  |  |
| 4.g3915 | KAF3976838 | hypothetical protein CMV_000012 | *Castanea mollissima* |  |  |  |  |  |  |
| 5.g2789 | XP_023898567 | uncharacterized protein LOC112010459 isoform X2 | *Quercus suber* | Q9M089_ARATH | Keratin-associated protein (DUF1218) |  |  | drought stress response | Xu, J., Yuan, Y., Xu, Y., Zhang, G., Guo, X., Wu, F., ... & Tang, Q. (2014). Identification of candidate genes for drought tolerance by whole-genome resequencing in maize. BMC Plant Biology, 14(1), 83. |
| 5.g3716 | XP_023870312 | dynamin-related protein 5A | *Quercus suber* | DRP5A_ARATH | Dynamin-related protein 5A | Probable microtubule-associated force-producing protein that is targeted to the forming cell plate during cytokinesis. May play a role in cell division | Miyagishima, S. Y., Kuwayama, H., Urushihara, H., & Nakanishi, H. (2008). Evolutionary linkage between eukaryotic cytokinesis and chloroplast division by dynamin proteins. Proceedings of the National Academy of Sciences, 105(39), 15202-15207. | drought stress response | Ren, Z., Zhang, D., Cao, L., Zhang, W., Zheng, H., Liu, Z., ... & Su, H. (2020). Functions and regulatory framework of ZmNST3 in maize under lodging and drought stress. Plant, Cell & Environment, 43(9), 2272-2286. |
| 5.g4697 | XP_023901205 | cytochrome b561 and DOMON domain-containing protein At4g12980-like | *Quercus suber* |  |  |  |  |  |  |
| 6.g180 | KNA23105 | hypothetical protein SOVF_026910 | *Spinacia oleracea* |  |  |  |  |  |  |
| 6.g3229 | XP_015867595 | transcription initiation factor TFIID subunit 11 | *Ziziphus jujuba* | TA14B_ARATH | Transcription initiation factor TFIID | Negative regulator of flowering controlling the H4K5 acetylation levels in the FLC and FT chromatin. Positively regulates FLC expression. | Zacharaki, V., Benhamed, M., Poulios, S., Latrasse, D., Papoutsoglou, P., Delarue, M., & Vlachonasios, K. E. (2012). The Arabidopsis ortholog of the YEATS domain containing protein YAF9a regulates flowering by controlling H4 acetylation levels at the FLC locus. Plant science, 196, 44-52. | drought stress response | Parvathi, M. S., Nataraja, K. N., Reddy, Y. N., Naika, M. B., & Gowda, M. C. (2019). Transcriptome analysis of finger millet (Eleusine coracana (L.) Gaertn.) reveals unique drought responsive genes. Journal of genetics, 98(2), 46. |
| 6.g823 | No hits found |  |  |  |  |  |  |  |  |
| 7.g1655 | XP_023909357 | histone deacetylase 6 | *Quercus suber* | HDA6_ARATH | Histone deacetylase 6 | Responsible for the deacetylation of lysine residues on the N-terminal part of the core histones (H2A, H2B, H3 and H4). |  | drought stress response | Kim, J. M., Sasaki, T., Ueda, M., Sako, K., & Seki, M. (2015). Chromatin changes in response to drought, salinity, heat, and cold stresses in plants. Frontiers in plant science, 6, 114. |
| 7.g2958 | KAF3966011 | hypothetical protein CMV_009858 | *Castanea mollissima* | F4I6B2_ARATH | SIT4 phosphatase-associated family protein |  |  |  |  |
| 7.g3483 | XP_023874962 | uncharacterized protein LOC111987473 | *Quercus suber* |  |  |  |  |  |  |
| 7.g3669 | XP_023883887 | protein trichome birefringence-like 38 | *Quercus suber* | TBL38_ARATH | protein trichome birefringence-like 38 | May act as a bridging protein that binds pectin and other cell wall polysaccharides. Probably involved in maintaining esterification of pectins |  | drought stress response | Shuai, P., Liang, D., Zhang, Z., Yin, W., & Xia, X. (2013). Identification of drought-responsive and novel Populus trichocarpamicroRNAs by high-throughput sequencing and their targets using degradome analysis. Bmc Genomics, 14(1), 233. |
| 8.g199 | KAF3962723 | hypothetical protein CMV_012798 | *Castanea mollissima* |  |  |  |  |  |  |
| 8.g2624 | No hits found |  |  |  |  |  |  |  |  |
| 8.g2764 | RUS78569 | hypothetical protein EGW08_013676, partial | *Elysia chlorotica* |  |  |  |  |  |  |
| 8.g2926 | RUS32681 | hypothetical protein BC938DRAFT_474617 | *Jimgerdemannia flammicorona* |  |  |  |  |  |  |
| 8.g3326 | XP_030953364 | sister chromatid cohesion protein SCC2 | *Quercus lobata* | SCC2_ARATH | sister chromatid cohesion protein SCC2 | Essential protein required for cell fate determination during embryogenesis | Sebastian, J., Ravi, M., Andreuzza, S., Panoli, A. P., Marimuthu, M. P., & Siddiqi, I. (2009). The plant adherin AtSCC2 is required for embryogenesis and sister‐chromatid cohesion during meiosis in Arabidopsis. The Plant Journal, 59(1), 1-13. |  |  |
| 8.g3872 | XP_030953755 | surfeit locus protein 2 | *Quercus lobata* | SURF1_ARATH | Surfeit locus protein 1 | Probably involved in the biogenesis of the COX complex |  |  |  |
| 8.g4454 | KAF3955069 | hypothetical protein CMV_019673 | *Castanea mollissima* |  |  |  |  |  |  |
| 8.g4554 | KAF3975054 | hypothetical protein CMV_001659 | *Castanea mollissima* |  |  |  |  |  |  |
| 8.g935 | KAF3972759 | hypothetical protein CMV_003766 | *Castanea mollissima* |  |  |  |  |  |  |
| 9.g1013 | KAF3963767 | hypothetical protein CMV_011877 | *Castanea mollissima* |  |  |  |  |  |  |
| 9.g1025 | XP_023873320 | cytosolic sulfotransferase 5-like | *Quercus suber* |  |  |  |  |  |  |
| 9.g2127 | No hits found |  |  |  |  |  |  |  |  |
| 9.g4398 | KAB5561320 | hypothetical protein DKX38_006277 | *Salix brachista* |  |  |  |  |  |  |
| 9.g4504 | KAF3976808 | hypothetical protein CMV_000062 | *Castanea mollissima* | TBL33_ARATH | Protein trichome birefringence-like 33 | Probable xylan acetyltransferase that plays a role in xylan acetylation and normal deposition of secondary cell walls | Yuan, Y., Teng, Q., Zhong, R., Haghighat, M., Richardson, E. A., & Ye, Z. H. (2016). Mutations of Arabidopsis TBL32 and TBL33 affect xylan acetylation and secondary wall deposition. PLoS One, 11(1), e0146460. | drought stress response | Shuai, P., Liang, D., Zhang, Z., Yin, W., & Xia, X. (2013). Identification of drought-responsive and novel Populus trichocarpamicroRNAs by high-throughput sequencing and their targets using degradome analysis. Bmc Genomics, 14(1), 233. |
| 9.g4548 | XP_023928861 | uncharacterized protein LOC112040195 | *Quercus suber* |  |  |  |  |  |  |
| 9.g4606 | XP_030928366 | LOW QUALITY PROTEIN: E3 ubiquitin-protein ligase SHPRH | *Quercus lobata* |  |  |  |  |  |  |
| 9.g653 | KAF3952878 | hypothetical protein CMV_021616 | *Castanea mollissima* |  |  |  |  |  |  |
| 10.g4171 | ONI05229 | hypothetical protein PRUPE_6G363600 | *Prunus persica* |  |  |  |  |  |  |
| 11.g2603 | XP_023892377 | amino acid permease 6-like | *Quercus suber* | AAP6_ARATH | Amino acid permease 6 | Amino acid-proton symporter. Stereospecific transporter with a broad specificity for tryptophan, proline, and neutral and acidic amino acids. | Rentsch, D., Hirner, B., Schmelzer, E., & Frommer, W. B. (1996). Salt stress-induced proline transporters and salt stress-repressed broad specificity amino acid permeases identified by suppression of a yeast amino acid permease-targeting mutant. The Plant Cell, 8(8), 1437-1446. | environmental stress response | Rentsch, D., Hirner, B., Schmelzer, E., & Frommer, W. B. (1996). Salt stress-induced proline transporters and salt stress-repressed broad specificity amino acid permeases identified by suppression of a yeast amino acid permease-targeting mutant. The Plant Cell, 8(8), 1437-1446. |
| 11.g787 | No hits found |  |  |  |  |  |  |  |  |
| 12.g1692 | KAF3972258 | hypothetical protein CMV_004214 | *Castanea mollissima* |  |  |  |  |  |  |
| 12.g2592 | No hits found |  |  |  |  |  |  |  |  |
| 12.g3049 | XP_023886583 | transcription factor TCP15 | *Quercus suber* | TCP15_ARATH | transcription factor TCP15 | Transcription factor involved the regulation of plant development. Together with TCP14, modulates plant stature by promoting cell division in young internodes. | Kieffer, M., Master, V., Waites, R., & Davies, B. (2011). TCP14 and TCP15 affect internode length and leaf shape in Arabidopsis. The Plant Journal, 68(1), 147-158. | environmental stress response | Viola, I. L., Camoirano, A., & Gonzalez, D. H. (2016). Redox-dependent modulation of anthocyanin biosynthesis by the TCP transcription factor TCP15 during exposure to high light intensity conditions in Arabidopsis. Plant Physiology, 170(1), 74-85. |
| 12.g922 | KAF3957893 | hypothetical protein CMV_017140 | *Castanea mollissima* |  |  |  |  |  |  |

**Supplementary file 1E**. List of 20 most informative SNPs as selected by the eSPA Analysis allowing for 85% correct classification.

|  | **scaffold** | **position** | **gene** | **swiss_prot_id_(arabidopsis_thaliana)** |
| --- | --- | --- | --- | --- |
| **41** | Fsyl.bwa_aln.clean.counts_GATC.12g6 | 1385435 | / | / |
| **56** | Fsyl.bwa_aln.clean.counts_GATC.12g7 | 31456694 | Fsyl.bwa_aln.clean.counts_GATC.12g7.g3617.t1 | Q9S7R3 |
| **14** | Fsyl.bwa_aln.clean.counts_GATC.12g12 | 7252002 | / | / |
| **9** | Fsyl.bwa_aln.clean.counts_GATC.12g11 | 21712586 | / | / |
| **42** | Fsyl.bwa_aln.clean.counts_GATC.12g6 | 1559115 | / | / |
| **43** | Fsyl.bwa_aln.clean.counts_GATC.12g6 | 1559277 | / | / |
| **44** | Fsyl.bwa_aln.clean.counts_GATC.12g6 | 1559370 | / | / |
| **27** | Fsyl.bwa_aln.clean.counts_GATC.12g2 | 22433752 | / | / |
| **58** | Fsyl.bwa_aln.clean.counts_GATC.12g7 | 33110000 | Fsyl.bwa_aln.clean.counts_GATC.12g7.g3816.t1 | Q94ID6 |
| **36** | Fsyl.bwa_aln.clean.counts_GATC.12g4 | 34077017 | Fsyl.bwa_aln.clean.counts_GATC.12g4.g3980.t1 | Q67YU0 |
| **84** | Fsyl.bwa_aln.clean.counts_GATC.12g9 | 38251152 | / | / |
| **85** | Fsyl.bwa_aln.clean.counts_GATC.12g9 | 38652380 | / | / |
| **59** | Fsyl.bwa_aln.clean.counts_GATC.12g8 | 1582675 | / | / |
| **1** | Fsyl.bwa_aln.clean.counts_GATC.12g1 | 10993370 | / | / |
| **78** | Fsyl.bwa_aln.clean.counts_GATC.12g9 | 20434572 | / | / |
| **82** | Fsyl.bwa_aln.clean.counts_GATC.12g9 | 37955715 | Fsyl.bwa_aln.clean.counts_GATC.12g9.g4504.t1 | F4IH21 |
| **80** | Fsyl.bwa_aln.clean.counts_GATC.12g9 | 25538827 | Fsyl.bwa_aln.clean.counts_GATC.12g9.g3080.t1 | Q9FMK9 |
| **34** | Fsyl.bwa_aln.clean.counts_GATC.12g4 | 33216309 | / | / |
| **70** | Fsyl.bwa_aln.clean.counts_GATC.12g9 | 5021966 | / | / |
| **69** | Fsyl.bwa_aln.clean.counts_GATC.12g8 | 38060740 | / | / |

**Supplementary file 1F**. Software pipeline and commands used for PoolSeq analysis.

#AUTOTRIM
perl ../autotrim-master/[autotrim.pl](http://autotrim.pl/) -fofn files.txt -trim ../trimmomatic_options -tt 12 -log /data/FagusPools -tp /home/mpfenninger/Trimmomatic-0.39/trimmomatic-0.39.jar -fqcp /home/mpfenninger/FastQC/fastqc

#Mapping with BWA

bwa index Beech_12Chr.masked.fas

bwa mem -t 12 -k 30 /Genome/Fagus_sylvatica_genome.fasta XXX_R1.autotrim.paired.fq XXX_R2_autotrim.paired.fq > XXX_bwamem.sam

while read poollist
do samtools view -b $poollist"_bwamem.sam" > $poollist".bam"
done < poollist
wait

samtools sort XX.bam > XX.sort.bam

#Remove Duplicates with Picard

java -jar /home/mpfenninger/Picard/picard.jar MarkDuplicates I=MPG.sort.bam O=MPG.sort.rmd.bam M=PoolMPG.dupstat.txt VALIDATION_STRINGENCY=SILENT REMOVE_DUPLICATES=TRUE

#Remove low quality mappings with SAMtools

samtools view -q 20 -@ 14 -f 0x0002 -F 0x0004 -F 0x0008 -b MPG.sort.rmd.bam > MPG.sort.rmd.q20.bam

samtools index MPG.sort.rmd.q20.bam

#Create pileup with mpileup

samtools mpileup -f /data/FagusPools/Genome/Fagus_sylvatica_genome.fasta -B -Q 0 MPG.sort.rmd.q20.bam MPK.sort.rmd.q20.bam SPG.sort.rmd.q20.bam SPK.sort.rmd.q20.bam > FagusPool.mpileup

#Convert mpileup to sync with Popoolation 2_1201

java -jar ~/popoolation2_1201/mpileup2sync.jar --input FagusPool.mpileup --output FagusPool.sync --fastq-type sanger --min-qual 20 --threads 14

#Filtering for indels
#get indels
perl ~/popoolation2_1201/indel_filtering/[identify-indel-regions.pl](http://identify-indel-regions.pl/) --indel-window 5 --input FagusPool.mpileup --output FagusPool.indels.gtf

#remove indels from sync
perl ~/popoolation2_1201/indel_filtering/[filter-sync-by-gtf.pl](http://filter-sync-by-gtf.pl/) --input FagusPool.sync --gtf FagusPool.indels.gtf --output FagusPool.idf.sync


#Fst calculation

perl ~/popoolation2_1201/[fst-sliding.pl](http://fst-sliding.pl/) --input FagusPool.idf.sync --output Fagus.fst --min-count 2 --min-coverage 15 --max-coverage 2% --pool-size 100 --window-size 1000 --step-size 1000

#Fisher's exact test with PoolSeq 0.35 in R
library("poolSeq")
Fagus <- read.sync(file="/data/FagusPools/FagusPool.idf.sync", gen=c(0,1,0,1), repl=c(1,1,2,2),  keepOnlyBiallelic=TRUE)
afMPG <- af(sync=Fagus, repl=1, gen=0)
afMPK <- af(sync=Fagus, repl=1, gen=1)
afSPG <- af(sync=Fagus, repl=2, gen=0)
afSPK <- af(sync=Fagus, repl=2, gen=1)

write.table(afMPG, "afMPG.txt", sep="\t")
write.table(afMPK, "afMPK.txt", sep="\t")
write.table(afSPG, "afSPG.txt", sep="\t")
write.table(afSPK, "afSPK.txt", sep="\t")

covMPG <- coverage(sync=Fagus, repl=1, gen=0)
covMPK <- coverage(sync=Fagus, repl=1, gen=1)
covSPG <- coverage(sync=Fagus, repl=2, gen=0)
covSPK <- coverage(sync=Fagus, repl=2, gen=1)

AFC_MP <- afMPG - afMPK
write.table(AFC_MP, "AFC_MP.txt", sep="\t")
AFC_SP <- afSPG - afSPK
write.table(AFC_SP, "AFC_SP.txt", sep="\t")

NA_MPG <- t(covMPG * afMPG)
Na_MPG <- covMPG - NA_MPG
NA_MPK <- t(covMPK * afMPK)
Na_MPK <- covMPK - NA_MPK

NA_SPG <- t(covSPG * afSPG)
Na_SPG <- covSPG - NA_SPG
NA_SPK <- t(covSPK * afSPK)
Na_SPK <- covSPK - NA_SPK

p.valuesMPmaxcov120 <- chi.sq.test(A0 = NA_MPG, a0 = Na_MPG, At = NA_MPK, at = Na_MPK, min.cov=15, min.cnt = 3, max.cov=120, log = TRUE)
quantile(p.valuesMPmaxcov120, probs = c(0.999, 0.9999, 0.99999), na.rm = TRUE, names = TRUE)
p.valuesSPmaxcov120 <- chi.sq.test(A0 = NA_SPG, a0 = Na_SPG, At = NA_SPK, at = Na_SPK, min.cov=15, min.cnt = 3, max.cov=120, log = TRUE)
quantile(p.valuesMPmaxcov120, probs = c(0.999, 0.9999, 0.99999), na.rm = TRUE, names = TRUE)

pdf("MPmaxcov120.pdf")
plot(p.valuesMPmaxcov120, main=paste0("MP"), ylim=c(0, max(p.valuesMPmaxcov120, na.rm=TRUE)), xlab="position", ylab="-log10(p)", pch=".")
dev.off()
pdf("SPmaxcov120.pdf")
plot(p.valuesSPmaxcov120, main=paste0("SP"), ylim=c(0, max(p.valuesMPmaxcov120, na.rm=TRUE)), xlab="position", ylab="-log10(p)", pch=".")
dev.off()

write.table(p.valuesMPmaxcov120, "p.valuestotmaxcov120.txt", sep="\t")
write.table(p.valuestotmaxcov120, "p.valuestotmaxcov120.txt", sep="\t")

#cmh-Test

covG <- t(coverage(sync=Fagus, repl=1:2, gen=0))
covK <- t(coverage(sync=Fagus, repl=1:2, gen=1))
A_G <- t(af(sync=Fagus, repl=1:2, gen=0)) * covG
a_G <- covG - A_G
write.table(A_G, "afG.txt", sep="/t")
A_K <- t(af(sync=Fagus, repl=1:2, gen=1)) * covK
a_K <- covK - A_K
write.table(A_K, "afK.txt", sep="/t")
p.valuestot <- cmh.test(A0 = A_G, a0=a_G, At=A_K, at=a_K, min.cov=15, max.cov=100, min.cnt=3, log=TRUE)
write.table(p.valuestot, "p.valuestot.txt", sep="\t")


p.valuestot <- cmh.test(A0 = A_G, a0=a_G, At=A_K, at=a_K, min.cov=15, max.cov=100, min.cnt=3, log=TRUE)
pdf("cmh.pdf")
plot(p.valuestot, main=paste0("cmh_fdr"), ylim=c(0, max(p.valuestot, na.rm=TRUE)), xlab="position", ylab="-log10(p)", pch=".")
dev.off()
quantile(p.valuestot, probs = c(0.999, 0.9999, 0.99999), na.rm = TRUE, names = TRUE)
write.table(p.valuestot, "p.valuestot.txt", sep="\t")
write.table(A_G, "afG.txt", sep="/t")
write.table(A_K, "afK.txt", sep="/t")
length(p.fdr[p.fdr > 4])

**Supplementary file 1G**. Workflow individual reseq GWAS.

####workflow beech GWAS individual reseq data######

####May 2020 Barbara Feldmeyer#######

autotrim v.0.6.1

bcftools v.1.9

gatk v.4.1.7.0

picard v.2.20.8

plink v.1.90b6.13

samtools v.1.10

1) trim reads with autotrim (https://github.com/schellt/autotrim)

2) index genome file

bwa index Fagus_sylvatica_genome_v2_masked.fasta

samtools faidx Fagus_sylvatica_genome_v2_masked.fasta

AND create dictionary

picard CreateSequenceDictionary.jar R=Fagus_sylvatica_genome_v2_masked.fasta

Step3a map reads to genome Fagus_sylvatica_genome_v2_masked.fasta

bwa mem -M -t 10 Fagus_sylvatica_genome_v2_masked.fasta $i_1_autotrim.paired.fq $i_2_autotrim.paired.fq | samtools sort -l 9 -O BAM -o ${FILES[$SLURM_ARRAY_TASK_ID]}.sort.bam"#

3b)MarkDuplicates

picard MarkDuplicates I=$i.sort.bam O=$i.sort.bam_marked_dup.bam M=$i.sort.bam_marked_dup_metrics.txt VALIDATION_STRINGENCY=SILENT REMOVE_DUPLICATES=true

3c) SortSAM

picard SortSam I=$i.sort.bam_marked_dup.bam O=$i.sort.bam_marked_dup.bam_sorted.bam VALIDATION_STRINGENCY=SILENT SORT_ORDER=coordinate

3d) index bam

picard BuildBamIndex INPUT=$i.sort.bam_marked_duplicates.bam_sorted.bam

3e) check mapping quality with qualimap

qualimap multi-bamqc -r -d qualimap_commands

==>> PF_001 very bad mapping quality (<1%!!!!) and skewed GC ratio in PF_050 => remove these two samples from further analyses

3f) create file with sample.bam specific read groups

sed 's/.*data\///' checkSize.xls | sed 's/\//\tRGSM=/' |sed 's/_BD.*HW/ RGID=HW/'| sed 's/_L/./'| sed 's/_[0-9].fq.gz/ RGLB=SOME RGPL=illumina RGPU=2/' > readgroups_beech.txt

3g) create batch file to run picard to modify and add readgroups to .bam file header

picard AddOrReplaceReadGroups I="$i.sort.bam_marked_duplicates.bam_sorted.bam" O="$i.sort.bam_marked_duplicates_sorted_rehead.bam" RGSM=$i RGID=HWTH2DSXX.4 RGLB=SOME RGPL=illumina RGPU=2" &

3h) index renamed bams

##########

#Round1

##########

4a)run HaplotypeCaller in GVCF mode

gatk HaplotypeCaller -I $i -O $i_haploCall.gvcf -R Fagus_sylvatica_genome.fasta -ERC GVCF

4b) combine using combineGVCF

gatk CombineGVCFs -R Fagus_sylvatica_genome_v2_masked.fasta --variant PF_002.sort.bam_marked_duplicates_sorted_rehead.bam_haploCall.gvcf --variant PF_003.bam_marked_duplicates_sorted_rehead.bam_haploCall.gvcf ... -O beech_cohort98indivs_R1.gvcf" &

4c) GenotypeGVCFs to jointly call Haplotypes

GenotypeGVCFs -R Fagus_sylvatica_genome_v2_masked.fasta -V beech_cohort98indivs_R1.gvcf -O beech_cohort98indivs_R1_genotyped.gvcf

4d) call and subset SNPs

gatk SelectVariants -V beech_cohort98indivs_R1_genotyped.gvcf --select-type-to-include SNP -O beech_cohort98indivs_R1_genotyped_SNPs.vcf

4e) create summary stats

bcftools stats beech_cohort98indivs_R1_genotyped_SNPs.vcf > beech_cohort98indivs_R1_genotyped_SNPs_summaryStats.txt

4f) hard-filter SNPs (we conduct this conservative hard filtration step since we don't have any pre-existing SNP set available to recalibrate SNPs)

gatk VariantFiltration -R Fagus_sylvatica_genome_v2_masked.fasta -V beech_cohort98indivs_R1_genotyped_SNPs.vcf -O beech_cohort98indivs_R1_genotyped_SNPs_hardfiltration.vcf --filter-expression 'QD < 2.0' --filter-name 'QD2' --filter-expression 'MQ < 50.0' --filter-name 'MQ50' --filter-expression 'MQRankSum < -12.5' --filter-name 'MQRankSum-12.5' --filter-expression 'ReadPosRankSum < -8.0' --filter-name 'ReadPosRankSum-8' --filter-expression 'FS > 80.0' --filter-name 'FS' --filter-expression 'SOR > 4.00' --filter-name 'SOR_4' --filter-expression 'QUAL < 10.0' --filter-name 'QUAL_10'

-grep and save passed variants

grep -E '^#|PASS' beech_ cohort98indivs_R1_genotyped_SNPs_hardfiltration.vcf > beech_cohort98indivs_R1_genotyped_SNPs_hardfiltrationPASS.vcf

4g) Baserecalibration

gatk --java-options "-Xmx4g" BaseRecalibrator -I $i -O fagus_recalR1_data.table -R Fagus_sylvatica_genome_v2_masked.fasta --known-sites beech_cohort98indivs_R1_genotyped_indels_hardfiltration.vcf --known-sites beech_cohort98indivs_R1_genotyped_SNPs_hardfiltration.vcf

4h) apply BQSR

gatk --java-options "-Xmx4g" ApplyBQSR -I $i -O $i_abqsr_R1.bam -R Fagus_sylvatica_genome_v2_masked.fasta --bqsr-recal-file fagus_recalR1_data.table

###########################

#Round 2

##############################

5a) R2_haplocaller

gatk --java-options "-Xmx4g" HaplotypeCaller -I $i -O $i_haploCall_R2.gvcf -R Fagus_sylvatica_genome_v2_masked.fasta -ERC GVCF

5b) R2_combine GVCF

gatk CombineGVCFs -R Fagus_sylvatica_genome_v2_masked.fasta --variant .... -O beech_cohort98indivs_R2.gvcf" &

5c) R2_GenotypeGVCFs

gatk --java-options "-Xmx250G" GenotypeGVCFs -R Fagus_sylvatica_genome_v2_masked.fasta -V beech_cohort98indivs_R2.gvcf -O beech_cohort98indivs_R2_genotyped.gvcf

5d) R2_call, select and subset SNPs

gatk SelectVariants -V beech_cohort98indivs_R2_genotyped.gvcf --select-type-to-include SNP -O beech_cohort98indivs_R2_genotyped_SNPs.vcf

create summary stats

bcftools stats beech_cohort98indivs_R2_genotyped_SNPs.vcf > beech_cohort98indivs_R2_genotyped_SNPs_summaryStats.txt

6) variant filtration

6a) Variants to table (Extract Variant Quality Score)

gatk --java-options "-Xmx250G" VariantsToTable R Fagus_sylvatica_genome_v2_masked.fasta -V beech_cohort98indivs_R2_genotyped_SNPs.vcf -F CHROM -F POS -F QUAL -F QD -F DP -F MQ -F MQRankSum -F FS -F ReadPosRankSum -F SOR -O cohort_all98beechSamples_genotyped_snp.table" &

6b)create diagnostic plots for Variants run skript in R following (https://evodify.com/gatk-in-non-model-organism/)

########################################

library('gridExtra')

library('ggplot2')

VCFsnps <- read.csv('cohort_all98beechSamples_genotyped_snp.table', header = T, na.strings=c("","NA"), sep = "\t")

VCFindel <- read.csv('cohort_all98beechSamples_genotyped_indel.table', header = T, na.strings=c("","NA"), sep = "\t")

dim(VCFsnps)

dim(VCFindel)

VCF <- rbind(VCFsnps, VCFindel)

VCF$Variant <- factor(c(rep("SNPs", dim(VCFsnps)[1]), rep("Indels", dim(VCFindel)[1])))

snps <- '#A9E2E4'

indels <- '#F4CCCA'

DP <- ggplot(VCF, aes(x=DP, fill=Variant)) + geom_density(alpha=0.3) +

geom_vline(xintercept=c(10,6200))

QD <- ggplot(VCF, aes(x=QD, fill=Variant)) + geom_density(alpha=.3) +

geom_vline(xintercept=2, size=0.7)

FS <- ggplot(VCF, aes(x=FS, fill=Variant)) + geom_density(alpha=.3) +

geom_vline(xintercept=c(60, 200), size=0.7) + ylim(0,0.1)

MQ <- ggplot(VCF, aes(x=MQ, fill=Variant)) + geom_density(alpha=.3) +

geom_vline(xintercept=40, size=0.7)

MQRankSum <- ggplot(VCF, aes(x=MQRankSum, fill=Variant)) + geom_density(alpha=.3) +

geom_vline(xintercept=-20, size=0.7)

SOR <- ggplot(VCF, aes(x=SOR, fill=Variant)) + geom_density(alpha=.3) +

geom_vline(xintercept=c(4, 10), size=1, colour = c(snps,indels))

ReadPosRankSum <- ggplot(VCF, aes(x=ReadPosRankSum, fill=Variant)) + geom_density(alpha=.3) +

geom_vline(xintercept=c(-10,10,-20,20), size=1, colour = c(snps,snps,indels,indels)) + xlim(-30, 30)

svg("cohort_98individuals_R2_beech20200609.svg", height=20, width=15)

theme_set(theme_gray(base_size = 18))

grid.arrange(QD, DP, FS, MQ, MQRankSum, SOR, ReadPosRankSum, nrow=4)

dev.off()

###########################

6c) VariantFiltration

gatk VariantFiltration -R Fagus_sylvatica_genome_v2_masked.fasta -V beech_cohort98indivs_R2_genotyped_SNPs.vcf -O beech_cohort98indivs_R2_genotyped_SNPs.vcf_filtered.vcf --filter-expression 'QD < 2.0' --filter-name 'QD2' --filter-expression 'MQ < 40.0' --filter-name 'MQ_40' --filter-expression 'SOR > 4.00' --filter-name 'SOR_4' --filter-expression 'QUAL < 10.0' --filter-name 'QUAL_10' --filter-expression 'FS > 60.0' --filter-name 'FS_60'

-grep and save passed variants

grep -E '^#|PASS' beech_cohort98indivs_R2_genotyped_SNPs.vcf_filtered.vcf > beech_cohort98indivs_R2_genotyped_SNPs.vcf_filteredPASSED.vcf

-stats

bcftools stats beech_cohort98indivs_R2_genotyped_SNPs.vcf_filteredPASSED.vcf

7) Plink association analysis

7.1. create phenotype file containing relevant phenoype information

beech_phenotype.phe

7.2. convert .vcf file to plink .ped format and keep biallic only

plink --vcf beech_cohort98indivs_R2_genotyped_SNPs.vcf_filteredPASSED_genotypeFiltered.vcf --biallelic-only strict --double-id --allow-extra-chr --set-missing-var-ids @:# --indep-pairwise 50 10 0.1 --out beech_cohort98indivs_SNPs

7.2b. additionally create a file with all sites, i.e. do not remove linked loci

plink --vcf beech_cohort98indivs_R2_genotyped_SNPs.vcf_filteredPASSED_genotypeFiltered.vcf --biallelic-only strict --double-id --allow-extra-chr --set-missing-var-ids @:# --make-bed --out beech_cohort98indivs_linkedSNPs

7.3. create PCA from linkage-pruned sites

-run plink to create PCA values

plink --vcf beech_cohort98indivs_R2_genotyped_SNPs.vcf_filteredPASSED_genotypeFiltered.vcf --double-id --allow-extra-chr --set-missing-var-ids @:# --extract beech_cohort98indivs_SNPs.prune.in --make-bed --pca --out beech_cohort98indivs_SNPs

-run R to plot the PCA

library(tidyverse)

pca<-read_table2("beech_cohort98indivs_SNPs.eigenvec", col_names=FALSE)

eigenval<-scan("beech_cohort98indivs_SNPs.eigenval")

pca <- pca[,-1]

names(pca)[1] <- "ind"

names(pca)[2:ncol(pca)] <- paste0("PC", 1:(ncol(pca)-1))

7.4. create missing stats

plink -bfile beech_cohort98indivs_SNPs --missing --out miss_stat_SNPs --allow-extra-chr

7.5. summary stats allele frequency

plink -bfile beech_cohort98indivs_SNPs --freq --out freq_stat_SNPs --allow-extra-chr

7.6. association analysis with automatic correction for multiple testing

plink --assoc --bfile beech_cohort98indivs_SNPs --allow-no-sex --adjust --allow-extra-chr
